# Chemical Diversity and Biological Activities of Essential Oils from *Licaria*, *Nectrandra* and *Ocotea* Species (Lauraceae) with Occurrence in Brazilian Biomes

**DOI:** 10.3390/biom10060869

**Published:** 2020-06-05

**Authors:** Júlia Karla A. M. Xavier, Nayara Sabrina F. Alves, William N. Setzer, Joyce Kelly R. da Silva

**Affiliations:** 1 Programa de Pós-Graduação em Química, Instituto de Ciências Exatas e Naturais, Universidade Federal do Pará, Belém 66075-900, Brazil; julia.xavier@icen.ufpa.br; 2 Programa de Pós-Graduação em Biotecnologia, Instituto de Ciências Biológicas, Universidade Federal do Pará, Belém 66075-900, Brazil; nayara.alves@icb.ufpa.br; 3 Department of Chemistry, University of Alabama in Huntsville, Huntsville, AL 35899, USA; wsetzer@chemistry.uah.edu; 4 Aromatic Plant Research Center, 230 N 1200 E, Suite 102, Lehi, UT 84043, USA

**Keywords:** sesquiterpenes, β-caryophyllene, α-bisabolol, antimicrobial, cytotoxic

## Abstract

Lauraceae species are known as excellent essential oil (EO) producers, and their taxa are distributed throughout the territory of Brazil. This study presents a systematic review of chemical composition, seasonal studies, occurrence of chemical profiles, and biological activities to EOs of species of *Licaria*, *Nectandra*, and *Ocotea* genera collected in different Brazilian biomes. Based on our survey, 39 species were studied, with a total of 86 oils extracted from seeds, leaves, stem barks, and twigs. The most representative geographic area in specimens was the Atlantic Forest (14 spp., 30 samples) followed by the Amazon (13 spp., 30 samples), Cerrado (6 spp., 14 samples), Pampa (4 spp., 10 samples), and Caatinga (2 spp., 2 samples) forests. The majority of compound classes identified in the oils were sesquiterpene hydrocarbons and oxygenated sesquiterpenoids. Among them, β-caryophyllene, germacrene D, bicyclogermacrene, caryophyllene oxide, α-bisabolol, and bicyclogermacrenal were the main constituents. Additionally, large amounts of phenylpropanoids and monoterpenes such as safrole, 6-methoxyelemicin, apiole, limonene, α-pinene, β-pinene, 1,8-cineole, and camphor were reported. *Nectandra megatopomica* showed considerable variation with the occurrence of fourteen chemical profiles according to seasonality and collection site. Several biological activities have been attributed to these oils, especially cytotoxic, antibacterial, antioxidant and antifungal potential, among other pharmacological applications.

## 1. Introduction

Lauraceae is one of the most primitive angiosperm families. It belongs to the subclass Magnoliidae and order Laurales [1]. Lauraceae species have the reputation of being difficult to identify because several collections are sterile or fruiting but lack the floral characters needed for identification [2]. This family of flowering species is widely distributed in regions of tropical and subtropical climates with more than 2500 species [3]. 

Brazil contains six areas of biomes: Amazon, Atlantic Forrest, Cerrado, Caatinga, Pantanal, and Pampa. The Amazon biome covers 49.3% of the Brazilian territory and has an extension of 4,199,249 km^2^ [4]. The Amazon has the largest tropical forest in the world, equivalent to one-third of the rainforest reserves, and is home to the greatest number of species of flora and fauna [4,5]. The Cerrado Biome is composed of both savanna and rural and forest formations [6]. Its plant formation occupies about 24% of the Brazilian territory and is the second-largest biome in extension, with an area of 2,036,448 km^2^ [4].

The Caatinga Biome occupies an area of about 10% of Brazil and has a territorial extension of 844,453 km^2^ [4]. The vegetation is characterized as shrub-shrub, comprising mainly low trees and shrubs, microfilaria, and some xerophytic characteristics [7,8]. The Atlantic Forest Biome is formed by a set of diverse forests, such as Ombrophilous Dense Forest, Mixed Ombrophilous Forest, Deciduous and Semideciduous Seasonal Forest, occupies about 13% of the Brazilian territory and 1,110,182 km^2^ of territorial extension [4,9].

In the extreme south of Brazil is the Pampa Biome, which occupies an area of approximately 176,496 km^2^ and about 2% of the national territory. It is predominantly rural vegetation, such as Planalto da Campanha, Depression Central, Planalto Sul-Rio-Grandense, and Plain Coastal [10,11]. The Pantanal Biome is considered one of the most humid and continuous regions on the planet and has the smallest territorial extension in Brazil (150,355 km^2^), occupying approximately 1.8%. As the types of vegetation in the Cerrado are predominant in this biome, vegetation similar to the Caatinga and small areas with forests also occurs [4].

In Brazil, Lauraceae species inhabit the biomes known as Caatinga, Cerrado and Pantanal, but the greatest biodiversity can be found in the Amazon and Atlantic biomes [12]. The family has approximately 439 species distributed in 24 genera in the country [12], and from these, around 240 species alone were found in the Amazon rainforest [13]. The genera *Ocotea*, *Nectandra*, and *Licaria*, are well-known for their timber since several species are employed to produce high-quality furniture [12,14]. The number of these species cataloged in Brazil is significant, and correspond to more than 50% of Brazilian Lauraceae taxa. The most representative genus is *Ocotea*, with 168 species followed by *Nectandra* (46 spp.) and *Licaria* (21 spp.) [13]. Despite the wide distribution of these genera in Brazil, few studies have focused on chemical composition and biological activities of their essential oils, which corresponds to only 15% of total species that are reported. The genus *Licaria* is characterized by species with double margin cup-shaped cupules and in some cases, with opposite leaves [2]. For example, the species *Licaria puchury-major* (Mart.) Kosterm, known as “puchury”, is native to the Brazilian Amazon, and its seeds are commonly used in folk medicine against stomach and intestinal diseases, insomnia and irritability [15,16]. In Borba, Brazil, the seeds are also employed with the tongue of a popular fish known as “pirarucu” to treat stomach troubles [17].

The group *Nectandra* has fruits placed in a cup-shaped cupule, and its tepals are spread at anthesis [2]. Infusion of *Nectandra megapotamica* (Spreng.) Mez leaves from Dourados, Brazil, are applied as a calmative agent and in the treatment of cough and the flu. Its shredded and heated barks are also used to treat furuncles [18]. The volatile oil of *Nectandra elaiophora* is used by native peoples from the Rio Negro and Rio Solimões, State of Amazonas, to treat eczema, psoriasis of the head, and to kill nits and lice [19].

The genus *Ocotea* is characterized by cupules of different sizes and shapes, varying from small and plate-like to cup-shaped forms. Tepals are erect or spreading at anthesis [2]. Fruits and seeds of *Ocotea diospyrifolia* (Meissn.) Mez are consumed as an aphrodisiac and used to warm the body and as a cold remedy and to treat hoarseness at Intervales State Park, São Paulo, Brazil [20]. The species *Ocotea odorifera* is popularly applied in Brazil to treat nervous system diseases, leucorrhea, edema, diarrhea [21], and dermatosis [22]. 

**Figure 1:**
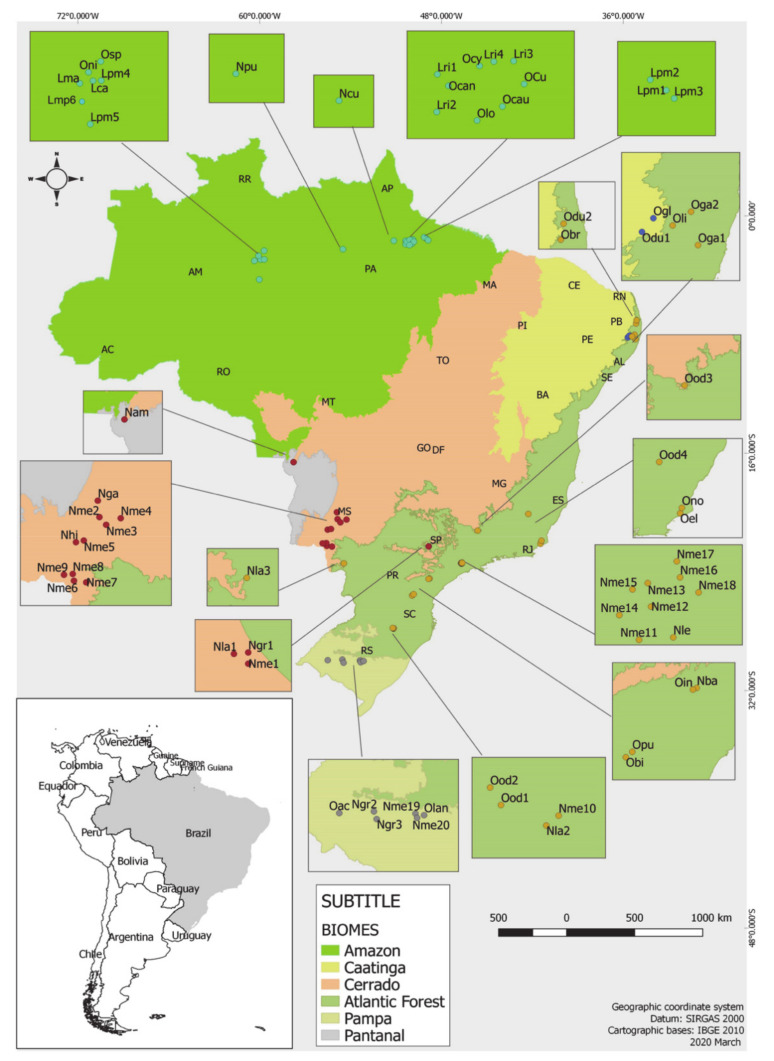
Geographical distribution in Brazilian biomes of *Licaria*, *Nectandra*, and *Ocotea* specimens based on its studies of essential oils. This map was built by the authors using the information of the collection site available in the bibliographic reference to each access. *Licaria canella*: (Lca); *L. martiniana*: (Lma), *L. puchury-major* (Lpm1, Lpm2, Lpm3, Lpm4, Lpm5, Lpm6); *L. rigida* (Lri1, Lr2, Lr3, Lri4), *Nectandra amazonum* (Nam), *N. barbellata* (Nba), *N. cuspidata* (Ncu), *N. gardneri* (Nga), *N. grandiflora* (Ngr1, Ngr2, Ngr3), *N. hihua* (Nhi), *N. lanceolata (*Nla1, Nla2, Nla3), *N. leucantha* (Nle), *N. megapotamica (*Nme1, Nme2, Nme3, Nme4, Nme5, Nme6, Nme7, Nme8, Nme9, Nme10, Nme11, Nme12, Nme13, Nme14, Nme15, Nme16, Nme17, Nme18, Nme19, Nme20), *N. puberula* (Npu), *Ocotea caniculata* (Ocan)*, O. caudata* (Ocau), *O. cujumary* (Ocu), *O. cymbarum* (Ocy), *O. duckei* (Odu1, Odu2)*, O. glomerata* (Ogl), *O. longifólia* (Olo), *O. nigrescen* (Oni), *O. splendens* (Osp)*, O. bicolor* (Obi), *O. bracteosa* (Obr), *O. elegans* (Oel), *O. indecora* (Oin)*, O. gardneri* (Oga1, Oga2), *O. limae* (Oli), *O. notata* (Ono), *O. odorífera* (Ood1, Ood2, Ood3, Ood4), *O. puberula* (Opu), *O. acutifólia* (Oac), *O. lancifolia* (Olan). Abbreviation list: AC: Acre; AL: Alagoas, AM: Amazonas, AP: Amapá, BA: Bahia, CE: Ceará, DF: Distristo Federal, ES: Espiríto Santo, GO: Goiás, MA: Maranhão, MT: Mato Grossso, MS: Mato Grosso do Sul, MG: Minas Gerais, PA: Pará, PB: Paraíba, PR: Paraná, PE: Pernambuco, PI: Piauí, RR: Roraíma, RO: Rondônia, RJ: Rio de Janeiro, RN: Rio Grande do Norte, RS: Rio Grande do Sul, SC: Santa Catarina, SP: São Paulo, SE: Sergipe, TO: Tocantins.

This study aimed to assemble the essential oil chemical compositions and their biological activities of the Lauraceae species that occur in Brazil. Based on our survey, there are reports on studies of EOs from 39 different species with geographical distribution according to Brazilian biome map (Figure 1). These species represented seventy-four accessions (specimens), totaling 86 samples of EOs obtained mainly from leaves, fruits, seeds, stem barks and twigs.

## 2. Distribution of Main Compound Classes in Essential Oil Samples

**Figure 2:**
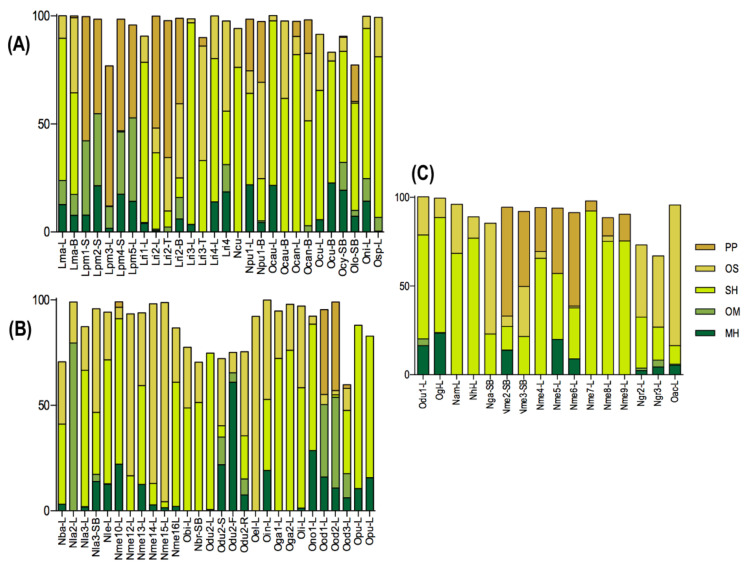
Distribution of compound class identified in essential oils from *Nectrandra*, *Licaria* and *Ocotea* species collected in Brazilian biomes. (**A**) EOs from Amazon: *Licaria martiniana* (Lma-L, Lma-S), *L. puchury-major* (Lpm1-S, Lpm2-S, Lpm3-L, Lpm4-S, Lpm5-L); *L. rigida*: (Lri1-L, Lr2-L, Lr3-L, Lri4-B), *Nectandra cuspidata* (Ncu-L), *N. puberula* (Npu-L, Npu-B), *Ocotea caudata* (Ocau-L, Ocau-B), *O. caniculata* (Ocan-L, Ocan-B), *O. cujumary* (Ocu-L, Ocu-B), *O. cymbarum* (Ocy-SB), *O. longifólia* (Olo-SB), *O. nigrescen* (Oni-L),), *O. splendens* (Osp-L); (**B**) EOs from biomes Caatinga, Cerrado and Pampa: *Ocotea duckei* (Odu1-L), *O. glomerata* (Ogl-L), *Nectandra amazonum* (Nam-L), *N. hihua* (Nhi-L), *N. gardneri* (Nga-SB), *N. megapotamica* (Nme2-SB, Nme3-SB, Nme5-L, Nme6-L, Nme7-L, Nme8-L, Nme9-L), *N. grandiflora* (Ngr2-L, Ngr3-L), O. *acutifólia* (Oac-L); (**C**) EOs from Mata Atlantic: *Nectandra barbellata* (Nba-L), *N. lanceolata* (Nla2-L, Nla3-L, Nla3-SB), *N. leucantha* (Nle-L), *N. megapotamica* (Nme10-L, Nme12-L, Nme13-L, Nme14-L, Nme15-L, Nme16-L), *Ocotea bicolor* (Obi-L), *O. bracteosa* (Obr-SB), *O. duckei* (Odu2-L, Odu2-S, Odu2-F, Odu2-R), *O. elegans* (Oel-L), *O. indecora* (Oin-L), *O. gardneri* (Oga1-L, Oga2-L), *O. limae* (Oli-L), *O. notata* (Ono-L), *O. odorífera* (Ood1-L, Ood2-L, Ood3-L), *O. puberula* (Opu-L, Opu-B). Abbreviation list: L: leaves, S: seeds, B: branch, T: twigs, SB: stem bark, F: fruits, R: roots. OS: oxygenated sesquiterpenoids, SH: sesquiterpene hydrocarbons, OM: oxygenated monoterpene, MH: monoterpene hydrocarbons, PP: Phenylpropanoids.

In this section, the oils were classified based on the percentage of the most abundant chemical compound class. Thus, the oils were found to be rich in monoterpene hydrocarbons, sesquiterpene hydrocarbons, oxygenated sesquiterpenoids, phenylpropanoids, and benzenoids. Some EO displayed the main compounds that belonged to different classes than the majority in the oils. For example, the oil of *Ocotea* bicolor Vattimo-Gil collected in Curitiba (PR, Brazil) exhibited a predominance of sesquiterpene hydrocarbons (48.77%). However, the phenylpropanoid dillapiole (15.2%) in combination with δ-cadinene (20.0%), α-cubebene (6.5%), and α-copaene (5.1%) were the main compounds. The distribution of compound classes, according to its respective biome, can be visualized in Figure 2.

## 3. Volatile Profiles 

### 3.1. Oils Rich in Monoterpene Hydrocarbons 

The oils of leaves from *Nectandra megapotamica* (Spreng.) Mez collected in Botucatu (SP, Brazil) presented high amounts of monoterpene hydrocarbons (52.2%), including α-pinene (18.37%) and β-pinene (16.65%) [23]. These amounts showed a variation according to leaves maturation stage to a specimen collected in Santa Maria (RS). The percentages of monoterpenes hydrocarbons were 46.3% and 51.3% to young and adult leaves, respectively. The major compounds were α-pinene (25.1–28.0%) and β-pinene (14.4–16.3%) [24].

### 3.2. Oils Rich in Sesquiterpene Hydrocarbons

As a class, the sesquiterpene hydrocarbons are very well represented in Lauraceae essential oils, especially the caryophyllane, humulane, germacrane, and selinane skeletons.

The oils of leaves and Stem of *L. martiniana* collected in Belém (PA) were rich in hydrocarbons sesquiterpenes with percentages of 65.8% and 47%, respectively. The compounds β-caryophyllene (41.70%) and β-selinene (7.90%) were the major constituents of the leaves, and β-caryophyllene (21.40%) in the stems [25]. The content of sesquiterpene hydrocarbons in the oils of specimens of *Licaria rigida* Kosterm. Kosterm collected in Melgaço (PA) varied from 66.34% to 93.33% [26,27]. Among them, two samples displayed β-caryophyllene (59.40–76.09%) and α-humulene (6.61%–7.80%) as the main compounds. However, another specimen showed δ-cadinene (10.53%), β-caryophyllene (9.73%) and β-bourbonene (9.44%) [27].

The EO of *Nectandra amazonum* Nees collected in Cáceres (MS, Brazil) showed high amounts of sesquiterpene hydrocarbons (68.4%) with β-caryophyllene (28.5%) and germacrene D (14.8%) the most representative [28]. The content of sesquiterpene hydrocarbons in the EO of *N. barbellata* Coe-Teixeira was 37.64%, and δ-cadiene (11.42%) and β-caryophyllene (9.79%) were the major compounds [3]. 

*Nectandra cuspidata* Nees & Mart. oil from a specimen collected in Melgaço (Amazon, Brazil) displayed a concentration of 76.2% sesquiterpene hydrocarbons; β-caryophyllene (26.9%) and bicyclogermacrene (16.0%) were dominant [29]. *Nectandra hihua* (Ruiz & Pav.) collected in Maracaju (MS, Brazil), displayed an oil dominated by bicyclogermacrene (28.1%), germacrene D (13.8%) and β-caryophyllene (9.0%). The total amount of sesquiterpene hydrocarbons was of 68.0% [28]. Sesquiterpene hydrocarbons displayed contents of 64.6% and 79.6% in specimens of *N. lanceolata* collected in Novo Mundo (MS, Brasil) and Barracão (RS, Brasil), respectively. For both samples, the main compounds were bicyclogermacrene (18.2%, 27.8%) and β-caryophyllene (12.45%, 32.5%) [30,31]. 

Compounds with the germacrane skeleton, such as bicyclogermacrene (33.4%) and germacrene D (16.8%), were predominant in the oil of *N. megapotamica* collected in Barracão (RS, Brasil). The content of sesquiterpene hydrocarbons was 79.60% [31]. In another study, the chemical composition during the different maturation stages of *N. megapotamica* collected in Santa Maria (RS) was evaluated. The oils showed a content of sesquiterpene hydrocarbons of 59.75% and 49.97% in young and adult plants, respectively. The main compounds identified were bicyclogermacrene (46.47%, 34.56%) and germacrene D (9.61%, 9.2%) [32]. In addition, bicyclogermacrene (28.44%) and germacrene A (7.34%) were the main compounds in the oil of leaves of *Nectandra leucantha* Nees & Mart collected in Cubatão (SP). The total of sesquiterpene hydrocarbons in this sample was 58.78% [33].

The oil of *Ocotea bicolor* Vattimo-Gil collected in Curitiba (PR, Brazil) exhibited a predominance of sesquiterpene hydrocarbons (48.77%) distributed in small percentages such as δ-cadinene (20.0%), β-sesquiphellandrene (6.67%) and β-elemene (5.41%) [34]. Likewise, the oil from the stem bark of *Ocotea bracteosa* (Meisn.) Mez collected in Santa Rita (PB) showed 52.1% of sesquiterpene hydrocarbons with a predominance of δ-cadinene (12.4%) and ledene (11.1%) [35].

High amounts of sesquiterpene hydrocarbons were identified in oils extracted from the leaves and the stems of five *Ocotea* species collected in Melgaço (PA). β-Selinene (20.3%, 12.1%), β-caryophyllene (18.9%, 7.1%) and 7-*epi*-α-selinene (14.3%, 9.0%) were the main compounds in the leaves and stems of *Ocotea caniculata* (Rich.), and their total percentages of sesquiterpene hydrocarbons were 82.1% and 48.6%, respectively [36]. *Ocotea caudata* (Nees) Mez displayed a total of 76.2% and 61.8% in the leaves and stems, respectively. In the leaf oil, bicyclogermacrene (29.6%) and germacrene D (19.9%) were the main compounds. However, the oil from the stems displayed δ-cadinene (13.8%), germacrene D (8.9%), and β-guaiene (8.3%) [36]. Sesquiterpene hydrocarbons represented a total of 59.8% in the leaves and 56.5% in the stems of *Ocotea cujumary* Mart. The leaf oil showed β-caryophyllene (22.2%) and δ-cadinene (6.6%) as major compounds, and in the stems were β-caryophyllene (8.1%), and germacrene D (5.9%) [36]. The oils extracted from stem barks of *Ocotea cymbarum* Aubl and *Ocotea longifolia* H.B.K showed the amounts of sesquiterpene hydrocarbons were 51.4% and 49.6%, respectively. The most abundant compounds were α-selinene (25.8%) and δ-cadinene (18.6%) in *O. cymbarum* and δ-cadinene (20.0%), α-cubebene (6.5%) and α-copaene (5.1%) in O. *longifolia* [26]. 

Sesquiterpene hydrocarbons showed percentages of 55.7% and 78.15% in EOs of individuals of *Ocotea duckei* Vattimo collected in Camocim de São Felix (PE) and Santa Rita (PB), respectively. β-Caryophyllene (18.1%) and valencene (17.6%) are the main compounds for the first one and β-caryophyllene (60.54%) for the second [37,38]. The EOs of two specimens of *Ocotea gardneri* (Meisn.) collected in Igarassu (PE) exhibited concentrations of sesquiterpene hydrocarbons of 72.26% and 76.10%, respectively. Germacrene D (26.9%, 26.96%) and bicyclogermacrene (21.7%, 20.73%) were the major components [39,40]. Leaf essential oils from *O. gardneri* exhibited 51.3% of sesquiterpene hydrocarbons with a predominance of β-caryophyllene (29.28%), germacrene D (7.1%) and α-humulene (5.5%). The specimen collection site was not reported [41]. Leaves of *Ocotea glomerata* (Nees) Mez collected in Camocim de São Felix (PE) had 64.8% of sesquiterpene hydrocarbons with a predominance of aromadendrene (17.3%) and β-caryophyllene (14.6%) [37].

The EOs of *Ocotea limae* Vattimo-Gil and *Ocotea notata* (Ness) Mez collected in Iguarassu (PE), and Carapebus (RJ) showed amounts of sesquiterpene hydrocarbons of 57.1% and 59.9%, respectively. Compounds with caryophyllane and germacrane skeletons were predominant such as β-caryophyllene (12.4%) and bicyclogermacrene (11.3%) in *O. limae* and β-caryophyllene (22.9%) and germacrene A (22.7%) in *O. notata* [39,42].

The oils of leaves and twigs of *Ocotea puberula* acollected in Curitiba (PR, Mata Atlântica) were rich in hydrocarbons sesquiterpenes with percentages of 77.4% and 67.22%, respectively. In the both samples, β-caryophyllene (31.0%, 14.0%), bicyclogermacrene (14.0%, 31.0%), and β-elemene (9.7%, 5.3%) were the main compounds [43]. Also, β-caryophyllene was the most abundant compound in the oils of *O. nigrescens* Vicentini (37.9%) and *O. splendens* (Meisn.) Baill (51.0%) both collected in Manaus (AM). The total amounts of sesquiterpene hydrocarbons exhibited values of 69.4% and 74.3%, respectively [44]. 

### 3.3. Oils Rich in Oxygenated Sesquiterpenoids

The EO of leaves of two specimens of *Nectandra grandiflora* Ness & Mart. ex Ness collected in Jaguari (RS), and a specimen collected Botucatu (SP) were abundant in oxygenated sesquiterpenoids with amounts of 40.71%, 40.08%, and 60.15%, respectively. For the first specimen, the main compound was dehydrofukinone (26.85%, 24.7%) and in the second was *iso*-bicyclogermacrenal (34.02%) and spathulenol (15.75%) [23,45,46]. The composition of *N. lanceolata* was very similar and displayed *iso*-bicyclogermacrenal (35.0%) and spathulenol (13.9%) as major components and a concentration of 52.57% of oxygenated sesquiterpenoids [23]. The EOs of four specimens of *N. megapotamica* collected in Atlantic Forest (SP) displayed high amounts of oxygenated sesquiterpenoids (70.3–94.5%). The main compound was α-bisabolol (59.7–93.7%) [47,48].

Caryophyllene oxide was the main compound of the EO from *Ocotea acutifolia* (Nees) Mez, and *O. lancifolia* (Schott) collected in São Francisco de Assis and Santa Maria (RS), respectively [49,50]. The oil of *O. acutifolia* was dominated by caryophyllene oxide (56.9%), and calarene epoxide (11.74%) and the specimens of *O. lancifolia* oils presented caryophyllene oxide (39.4–46.4%) and *allo*-himachallol (5.7–8.0%). Total amounts of oxygenated sesquiterpenoids in both displayed an average of 79.25% [49,50]. Besides, other tissues of *O. lancifolia* also had high levels of oxygenated sesquiterpenoids such as the inflorescences (81.3%) and fruits (69.1%). Once again, caryophyllene oxide (27.9–52.1%) was the most abundant compound [50].

*O. duckei* showed a predominance of oxygenated sesquiterpenoids in oils extracted from stems and roots with contents of 31.76 and 39.76%, respectively. β-Eudesmol (27.51%) was the main compound in the stems, and the oil of roots showed elemol (24.31%) and β-eudesmol (13.44%) [38]. Conversely, the leaf oil of *Ocotea elegans* Mez collected in Carapebus (RJ) displayed a high amount of sesquirosefuran (92.20%) [51].

### 3.4. Oils Rich in Sesquiterpene Hydrocarbons and Oxygenated Sesquiterpenes

The amounts of sesquiterpene hydrocarbons and oxygenated sesquiterpenoids in oils from the leaves of *N. megapotamica* collected in São Paulo (SP) were 46.9%, 58.90% and 25.7%, 34.5%, respectively. The main compounds were: *iso*-spathulenol (26.8%), δ-elemene (23.8%) and β-bisabolene (13.3%); β-sesquiphellandrene (32.0%), β-bergamotene (19.0%) and α-bisabolol (8.9%) [23]. The oils of *Ocotea indecora* (Shott) Mez collected in Ribeirão Grande (SP) showed oxygenated sesquiterpenoids (47.18%) and sesquiterpene hydrocarbons (33.66%) in the leaves, and the main compounds were bicyclogermacrene (29.79%) and valerianol (15.12%) [3]. 

### 3.5. Oils Rich in Phenylpropanoids and Monoterpenes

The composition of *Licaria puchury-major* Mart. collected in Borba (AM) was rich in phenylpropanoids (43.0%) and oxygenated monoterpenoids (38.6%) being safrole (39.4%) and 1,8-cineole (27.6%) the main constituents [52]. The oils of two individuals of *Ocotea odorifera* (Vell.) collected in Marcelino Ramos (RS) showed phenylpropanoids (40.23%, 42.0%), oxygenated monoterpenoids (34.35%, 43.0%) and monoterpene hydrocarbons (16.1%, 10.8%). Safrole (42.0%, 40.23%), camphor (43.0%, 34.35%), camphene (6.0%, 5.0%) and limonene (7.42%, 3.0%) were the most representative compounds [53,54].

### 3.6. Oils Rich in Phenylpropanoids and Sesquiterpenes

The oil of *L. rigida* sampled in Melgaço (PA) revealed a high concentration of phenylpropanoids (51.86%), sesquiterpene hydrocarbons (35.42%), and oxygenated sesquiterpenoids (11.44%). The main compounds were 6-methoxy-elemicin (51.86%), β-caryophyllene (15.32%), and selin-11-en-4α-ol (9.68%) [27]. Phenylpropanoids (38.1%) and sesquiterpene hydrocarbons (30.0%) dominated the oil of *O. odorifera* leaves collected in Machado (MG). The most representative compounds were safrole (36.3%) followed by γ-cadinene (6.6%) [55].

The oils of *N. puberula* collected in Santarém (PA) showed contents of sesquiterpene hydrocarbons (42.4%) and phenylpropanoids (28.1%) in the leaves, and the main compounds were apiole (22.2%) and β-caryophyllene (15.1%). However, in the oil from the stems, the composition was characterized by oxygenated sesquiterpenoids (44.7%) and phenylpropanoids (28.1%). Apiole (28.1%), pogostol (19.8%), and guaiol (11.2%) were the main compounds [29].

### 3.7. Oils Rich in Benzenoids 

The chemical composition of the oil from the leaves of *Licaria canella* (Meissn.) Kosterm collected in Manaus (AM) showed a predominance of benzyl benzoate (71.35%) [56]. 

## 4. Occurrence of Different Chemical Profiles

The chemical composition varies among specimens of the same species of *Licaria*, *Nectandra* and *Ocotea*; the oils and the combination were characterized by their chemical profiles, which are based on the concentrations of the major components. These different chemical profiles may be associated with respect to ecological and geographical condition, age of plant and time of harvesting [24,32,50].

Studies on the chemical composition of EO from the leaves of *N. megapotamica* showed the occurrence of essential oils with different chemical profiles rich in bicyclogermacrene followed by terpenes such as α-pinene, β-pinene, germacrene D, limonene, elemene and lesser quantities of phenylpropanoids such as elemicin and asarone [31,32,48]. From individuals collected in the Rio Grande do Sul State, the occurrence of two different chemical profiles was observed. The first profile is represented by a sample from Santa Maria (RS, Pampa) and presented bicyclogermacrene, (46.47%, 34.56%), α-pinene (26.82%, 26.19%), germacrene D (9.61%, 9.20%) and β-pinene (7.95%, 12.3%) in the young and mature leaves, respectively (profile I) [32]. However, a specimen sampled in Barracão (RS, Atlantic Forest) displayed bicyclogermacrene (33.4%), germacrene D (16.8%) and limonene (14.1%) as main compounds (profile II) [31].

Although bicyclogermacrene (33.4%) was the main compound in oils of *N. megapotamica* from Atlantic Forest, some differences to EO from Mato Grosso do Sul state (MS, Cerrado) were observed. For the specimen collected in Macaraju, bicyclogermacrene (66.7%), germacrene D (18.2%), and elemicin (5.6%) were the main compounds (profile III). However, two individuals collected in Ponta Porã exhibited similar profiles rich in δ-elemene (32.2%, 37.9%), bicyclogermacrene (28.2%, 26.3%) and (*E*)-asarone (10.3%, 15.0%) (profile IV) [48]. Also, the oils from Campo Grande were classified into three profiles defined by sesquiterpene hydrocarbons (28.8–65.6%) and phenylpropanoids (24.8–52.7%). The main compounds for each profile were (*E*)-asarone (22.6%), δ-elemene (15.6%) and α-santalene (11.8%) (profile V); elemicin (35.9%), bicyclogermacrene (24.8%) and δ-3-carene (10.9%) (profile VI); elemicin (52.7%), bicyclogermacrene (8.9%) and α-pinene (5.7%) (profile VII) [48]. 

The literature reported the occurrence of at least seven additional profiles of EO of *N. megapotamica* collected in São Paulo State (Atlantic Forest, Brazil). The oxygenated sesquiterpenoid α-bisabolol was predominant, and its concentration varied from 66.05 to 93.7% [47,48]. The main compounds of each profile were: α-bisabolol (66.05%), δ-elemene (17.37%) and β-pinene (2,15%) (profile VIII) [47]; α-bisabolol (59.7%), δ-elemene (13.8%) and *iso*-spathulenol (11.3%) (profile IX); α-bisabolol (84.3%), germacrene D (4.0%) and β-bisabolene (2.5%) (profile X); α-bisabolol (93.7%), (*Z*)-β-ocimene (1.5%) and germacrene D (1.4%) (profile XI) [48]. Other profiles were represented by high amounts of monoterpene hydrocarbons (52.2%), sesquiterpene hydrocarbons (46.9–58.9%) and oxygenated sesquiterpenoids (25.7–34.5%). The most abundant compounds in the oils were *iso*-spathulenol (26.8%), δ-elemene (23.8%) and β-bisabolene (13.3%) (profile XII); β-sesquiphellandrene (32.0%), β-bergamotene (19.0%) and α-bisabolol (8.9%) (profile XIII); α-pinene (18.37%), β-pinene (16.65%) and bicyclogermacrene (10.8%) (profile XIV) [23,48]. 

The oils from stem barks of specimens of *N. megapotamica* collected in Campo Grande (MS, Cerrado) exhibited variation in chemical composition. The amounts of phenylpropanoids (61.4%, 42.3%), sesquiterpene hydrocarbons (13.3%, 21.5%), oxygenated sesquiterpenoids (5.8%, 28.2%), and monterpene hydrocarbons (13.8%, 0.0%) varied according to the collection site. The main compounds in the oil of specimens collected from a wet site were elemicin (41.7%), (*E*)-asarone (19.7%), and α-pinene (8.5%) (profile I). However, the plant collected in a dry site showed (*E*)-asarone (42.4%), α-cadinol (14.4%), and τ-cadinol (8.1%) (profile II) as the main constituents [28].

The chemical profiles of the EOs from the leaves of *N. lanceolata* showed variations according to the biomes from which they had been collected. The oils collected in the Pampa (Barracão, RS) and Cerrado (Novo Mundo, MS) showed similarity with a predominance of sesquiterpene hydrocarbons (79.6%, 64.6%) and oxygenated sesquiterpenoids (19.4%, 20.7%). The main compounds were β-caryophyllene (32.5%, 12.45%), bicyclogermacrene (27.8%, 18.2%) and spathulenol (11.80%, 16.7%) (profile I) [30,31]. The oil from the Atlantic Forest (Botocatu, SP) was characterized by oxygenated sesquiterpenoids (52.57%), *iso*-bicyclogermacrenal (35.0%) and spathulenol (13.9%), and a sesquiterpene hydrocarbon, β-selinene (7.0%) (profile II) [23].

The main compounds of *N. grandiflora* from Atlantic Forest and Pampa biomes were oxygenated sesquiterpenoids (60.17% and 40.71%, respectively). The major constituents identified in the sample collected in Botocatu (SP) were identified as *iso*-bicyclogermacrenal (34.0%), spathulenol (15.75%), and rosadiene (13.65%) (profile I) [23]. Meanwhile, the presence of dehydrofukinone (26.85%), valencene (6.89%), and the diterpene kaurene (6.03%) characterized the oils from Jaquari (RS) (profile II); dehydrofukinone (24.70%), bicyclogermacrene (5.93%), and kaurene (5.49%) (profile III) [45,46]. 

Two profiles of *O. duckei* oils were characterized by the presence of β-caryophyllene as the main constituent. The EO from the leaves of the specimen from Santa Rita (Atlantic Forest biome, PB) showed high content of β-caryophyllene (60.54%), followed by minor amounts of α-humulene (4.63%), and δ-selinene (4.4%) (profile I) [38]. The profile reported for the oil of a specimen collected in Camocim de São Félix (Caatinga biome, PE) was rich in β-caryophyllene (18.1%), valencene (17.6%) and elemol (6.8%) (profile II) [37].

There are two profiles of EOs from the leaves of *O. odorifera* collected in Atlantic Forest biome. The specimen collected in Machado (MG) showed safrole (36.3%) to be the main compound followed by low amounts of γ-cadinene (6.6%) and camphor (6.5%) (profile I) [55]. Likewise, camphor (43.0%, 34.35%), safrole (42.0%, 42.0%), and camphene (6.0%, 5.02%) were the major compounds of profile II from the city Marcelino Ramos (RS) [53,54]. 

Based on the literature, the oils from the leaves of *L. rigida* collected in Amazon (Melgaço, PA) can be classified into three profiles with β-caryophyllene, the most frequent compound. Two profiles are rich in sesquiterpenes with caryophyllane skeleton such as β-caryophyllene (59.40–76.09%), α-humulene (6.61–7.80%) and caryophyllene oxide (12.10%) (profile I) [26,27]; δ-cadinene (10.53%), β-caryophyllene (9.73%) and β-bourbonene (9.44%) (profile II) [27]. In contrast, the profile III had exhibited high amounts of 6-methoxyelemicin (51.86%), a phenylpropanoid, and the sesquiterpenoids β-caryophyllene (15.32%) and selin-11-en-4α-ol (9.68%). In addition, the oils from twigs and branches of these specimens displayed two profiles. Caryophyllene oxide (29.88%), 14-hydroxy-9-*epi*-β-caryophyllene (10.28%) and β-caryophyllene (8.92%) (twigs, profile I) and 6-methoxyelemicin (63.31%) and selin-11-en-4α-ol (23.99%) (twigs, profile II). Meanwhile, the main compounds presented in the branches were δ-cadinene (12.04%), terpinen-4-ol (10.67%) and selin-11-en-4α-ol (7.67%) (profile I) and 6-methoxyelemicin (39.55%) and selin-11-en-4α-ol (21.82%) (profile II) [27].

The EO extracted from seeds of various samples of *L. puchury-major* collected in Belém (PA) showed similar chemical profile rich in phenylpropanoids (43.81–57.50%) distributed in two profiles. The main compounds of the two samples were safrole (51.3%), 1,8-cineole (25.5%), α-terpineol (8.60%) (profile I), safrole (38.80%, 36.11%), 1,8-cineole (21.70%, 21.12%) and limonene (8.27%, 12.2%) (profile II) [15,16,57]. However, the EO of seeds collected in Manaus (AM, Amazon) displayed safrole (58.4%), dodecanoic acid (13.7%) and α-terpineol (8.4%) (profile III) [58].

## 5. Seasonal Variation in the Volatile Constituents

**Figure 3:**
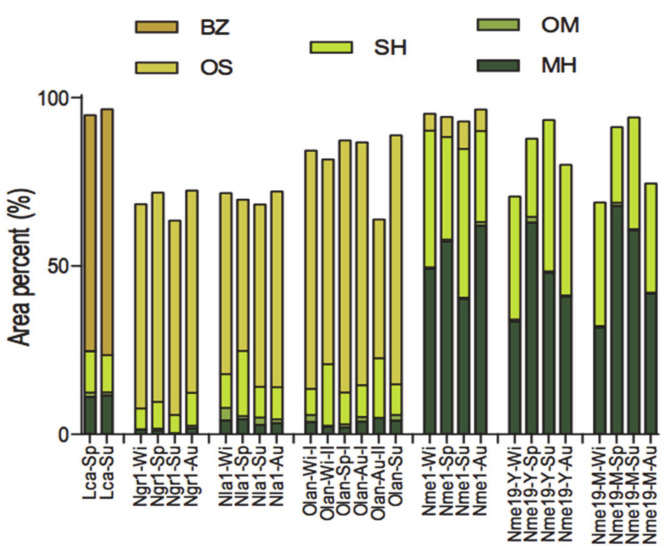
Variations in compounds classes in EO from the leaves of Lauraceae species during seasonal studies. EOs from Amazon: *Licaria canella* (Lca-Sp, Lca-Su); Cerrado: *Nectandra grandiflora* (Ngr1-Wi, Ngr1-Sp, Ngr1-Su, Ngr1-Au), *N. lanceolata* (Nla1-Wi, Nla1-Sp, Nla1-Su, Nla1-Au); Pampa: *Ocotea lancifolia* (Olan-Wi-I, Olan-Wi-II, Olan-Wi-Sp, Olan-Au-I, Olan-Au-II, Olan-Su); Cerrado: *N. megapotamica* (Nme1-Wi, Nme1-Sp, Nme1-Su, Nme1-Au); Pampa: *N. megapotamica* (Nme19-Y-Wi, Nme19-Y-Sp, Nme19-Y-Su, Nme19-Y-Au; Nme19-M-Wi, Nme19-M-Sp, Nme19-M-Su, Nme19-M-Au). Abbreviation list: Sp: spring, Su: summer, Wi: winter, Au: autumn; Y: young, M: mature; BZ: benzenoid, OS: oxygenated sesquiterpenoids, SH: sesquiterpene hydrocarbons, OM: oxygenated monoterpene, MH: monoterpene hydrocarbons.

Several studies on Lauraceae species have shown that changes in the chemical composition and yield of EO can be affected by humidity, temperature, seasonality, luminosity, photoperiod, geographic variations, plant age, tissue collected and phenologic stages [24,47,50]. The variations in the chemical composition in the oil from the leaves presented in this study are illustrated in Figure 3.

The seasonality and phenological aspects influenced in the EO production of *N. megapotamica* can probably be attributed to morphological parameters such as alterations in the leaves and metabolites due to environmental adaptation (pollinator attraction, seed dispersers, defense against herbivory and pathogens). Juvenile and mature leaves of *N. megapotamica* were collected in the city Morro do Elefante (Santa Maria, RS, Brasil) during the different seasons. Leaves collected in the spring, the season that includes flowering, fruiting, and foliation, displayed the higher EO yield with a percentage of 0.59% and 0.30% in juvenile and mature leaves, respectively. The range of leaf oil yield was lower (0.21–0.28%) in the autumn, the period in which the plant is in vegetative and reproductive rest, and of abscission of the vegetal organs for the winter [24]. 

EO chemical composition showed no influence on stage maturity on the leaves. The main compound classes were monoterpene hydrocarbons (47.0%, 51.8%) and sesquiterpene hydrocarbons (35.9%, 31.2%) represented by α-pinene (25.1%, 28.0%), bicyclogermacrene (24.6%, 22.3%) and β-pinene (14.4%, 16.3%). However, according to climatic changes, quantitative variations were observed. α-Pinene production was higher in the spring (33.23%), while the bicyclogermacrene amounts increased in the summer (32.93%) and decreased in the autumn (26.86%) and winter (23.10%). In the mature leaves, α-pinene was the main compound in all seasons (36.86–24.86%), excepted in the winter; there was a higher production of bicyclogermacrene (23.6%) [24].

Oxygenated sesquiterpenes represented the majority class in the leaves EO of *N. megapotamica*, collected in São Paulo City during the summer and winter. In the summer, the amounts of oxygenated sesquiterpenoids and sesquiterpene hydrocarbons were 70.3% and 11.95%, respectively. However, in the winter, the percentage of oxygenated sesquiterpenoids decreased to 64.5%, and sesquiterpene hydrocarbons increased to 22.5%. In both seasons, the main compounds were α-bisabolol and δ-elemene, in the summer (68.55%, 12.2%) and winter (63.55%, 22.55%). In addition, the monoterpene hydrocarbons were identified in lower percentages, as α-pinene (2.65%) and β-pinene (2.6%) in the winter, and safrole, a phenylpropanoid (1.45%) in the summer [47].

The seasonal changes influenced the oil yield and chemical composition of leaves EO of *N. lanceolata*, *N. grandiflora*, and *N. megapotamica* collected in Botucatu (São Paulo, Brazil). The EO yields of *N. lanceolata* and *N. grandiflora* were constant with values of 0.23%, 0.17% (spring) and 0.20%, 0.17% (autumn). For both species, the lower yields (<0.10%) were observed in samples collected in the winter [23]. The oxygenated sesquiterpenes represented the main compound class in the *N. grandiflora* EO with the higher and lower amounts in the spring (62.2%) and summer (57.7%), respectively. The main compounds were *iso*-bicyclogermacrenal (27.8–38.6%), spathulenol (11.1–20.1%), and rosadiene (11.2–15.1%) during all seasons [23]. Similarly, the chemical profile of de *N. lanceolata* EO was represented by oxygenated sesquiterpenoids with a higher percentage in the autumn (58.2%) and lower in the winter (44.9%). *iso*-Bicyclogermacrenal (27.8–39.6%) was the main compound in all seasons, followed by spathulenol (11.9–20.2%) and bicyclogermacrene (5.5–4.8%). In the winter, the spathulenol level decreased to 7.6%, and there was an increase of bicyclogermacrene (12.6%) [23].

The oil yield of *N. megapotamica* showed an average of 0.036% during the seasons. In the autumn, winter, and spring, the concentrations of monoterpene hydrocarbons were higher (62.0%, 49.2%, 57.2%) in comparison to sesquiterpene hydrocarbons (27.0%, 40.6%, 30.4%). However, in the summer, the higher and lower amounts of monoterpene hydrocarbons and sesquiterpene hydrocarbons were observed (40.3% and 44.2%, respectively). The main compounds identified in the autumn and winter were α-pinene (25.1%, 20.1%), β-pinene (22.3%, 18.5%), and bicyclogermacrene (9.1%, 10.6%). In the spring, the major compounds were α-pinene (18.2%), β-pinene (16.2%), and α-phellandrene (10.0%). However, bicyclogermacrene (14.8%) and α-phellandrene (11.0%) were the major compounds in the summer, and the amounts of α-pinene and β-pinene decreased to 10.1% and 9.6% [23].

The chemical composition and yield of EOs of leaves, fruits, and inflorescences of *O. lancifolia* collected in the district of Santo Antão (Santa Maria, RS) were evaluated according to climate changes during a year. Oxygenated sesquiterpenoids were predominant during all periods in the leaves (79.2%), inflorescences (81.3%), and in the fruits (69.1%). A variation of chemical composition and oil yield was observed in the samples collected between August and November and in the period from May to July. These periods are related to ripening and attack by pathogens in plants [50]. A higher yield from the leaf EOs was observed in the spring (1.03%) and the summer (0.96%) in contrast to those obtained in the winter (0.56%) and autumn (0.6%). The lowest EO production per month was observed in May (0.27%) and July (<0.1%). Caryophyllene oxide (46.4–36.4%), bicyclogermacrene (7.8–6.1%), and *allo*-himachalol (8.0–5.7%) were the main compounds, except in May and July, which presented β-chenopodiol (20.9%, 17.4%), (*Z*)-nerolidyl acetate (9.3%, 8.7%) and kaurene (11.9%, 17.1%) [50]. 

The EO of inflorescences was extracted only during the autumn in April and May, and it displayed a yield of 2.49% and 0.55%, respectively. The major compounds were caryophyllene oxide (34.9%), bicyclogermacrene (8.1%), and atractylone (4.9%) in April, and β-chenopodiol (38.7%), α-guaiene (6.0%) and (*Z*)-nerolidyl acetate (4.5%) in May. Concerning the fruits, the collection period occurred in July (winter) and November (spring). September showed the higher oil yield (1.58%), which corresponds to the period that the fruits appear green and immature. The lowest EO content was observed in July, and after the maturation stage of the fruit in November (0.34%). β-Chenopodiol (17.1%), (*E*)-β-ocimene (6.2%), and γ-muurolene (4.7%) were the major compounds identified in July. In the intermediated period of fruit maturation (August to October), the oils were rich in caryophyllene oxide (52.1–46.2%), bicyclogermacrene (8.9–6.7%) and (*E*)-β-ocimene (2.8–3.1%). The mature fruits collected in November showed a decrease of caryophyllene concentration (27.9%), followed by bicyclogermacrene (6.90%) and *allo*-himachalol (6.70%) [50].

The EOs of the leaves of *L. canella* sampled in the Adolpho Ducke Forest Reserve (Manaus, AM, Brasil) were extracted during the dry season (October, spring) and the rainy season (February, summer). The rainy period exhibited a higher yield period (1.3%) in comparison to the dry period (1.20%). However, the chemical profile to both oils was similar showing high amounts of benzenoid compounds (71.3%, 74.9%). The main compound was benzyl benzoate (69.7%, 73.0%), followed by α-copaene (4.99%, 4.51%) and α-phellandrene (4.2%, 3.3%) in minor proportions [56].

The oil yield from the leaves *N. grandiflora* and different tissues of *O. odorifera* showed significant variation according to seasonality. The collection sites for the samples were Jaguari (RS) and Viçosa (MG), respectively [59,60]. *N. grandiflora* displayed higher EO production during the spring (0.75%) and the lower yield in the winter (0.39%) [59]. Regarding *O. odorifera* oils, the higher EO production was observed in the summer for leaves (0.86%) and during the spring for twigs (0.9%) and bark (1.37%) [60]. These studies did not report information on EO chemical composition, however. 

The information on the main compounds of EOs extracted from each tissue of Licaria, Nectandra, and Ocotea species, their corresponding collection data, and their extraction method are present in the Table 1. 

## 6. Biological Activities

**Figure 4:**
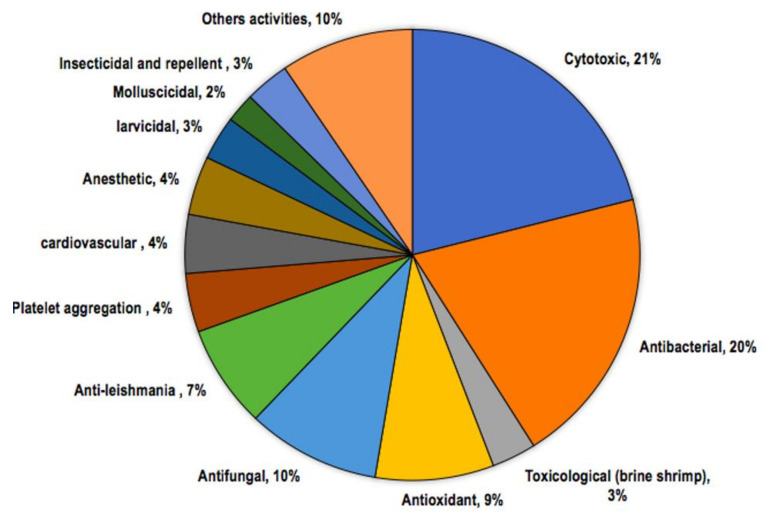
Distribution of studies on biological activities of EO from *Licaria*, *Nectandra* and *Ocotea* specimens with occurrence in Brazil.

All of the studies on biological activities of EOs of *Licaria*, *Nectandra*, and *Ocotea* species collected in Brazil corresponded to a total of 60 oils. Among them, six samples had no chemical composition reported. Several oils presented more than one specific activity, and the most frequent were cytotoxic, antibacterial, antioxidant, and antifungal activities. The percentages of the reported bioactivities and details of biological assays are present in Figure 4 and Table 2, respectively. 

### 6.1. Antibacterial Activity

The antibacterial activity of several species was evaluated by the disc diffusion method. The leaf EO of *O. odorifera* collected from Marcelino Ramos (RS) were tested against seventeen bacterial strains: *Enterococcus faecalis*, *Micrococcus luteus*, *Sarcina* sp., *Staphylococcus aureus*, *Staphylococcus epidermidis*, *Streptococcus mutans* (Gram-positive) and *Acinetobacter* sp., *Aeromonas* sp., *Citrobacter freundii*, *Escherichia coli*, *Klebsiella pneumoniae*, *Proteus mirabilis*, *Proteus vulgaris*, *Salmonella choleraesuis*, *Serratia marcescens*, *Shigella flexneri* and *Yersinia enterocolitica* (Gram-negative). The oil was tested at volumes varying from 5.0 to 20.0 μL where chloramphenicol (30.0 μg) was used as positive control. EO major components were safrole (40.23%), camphor (34.35%) and limonene (7.42%). In general, a higher potential was observed for Gram-negative (8.40–15.40 mm) than for Gram-positive bacteria (7.90–11.80 mm). Unfortunately, minimum inhibitory concentrations (MIC) were not determined [54]

Furthermore, leaves of *L. puchury-major* from Borba (AM) were tested against *Pseudomonas aeruginosa*, *E. coli*, *Streptococcus agalactiae* and *S. aureus*. This species of EO was composed mainly of safrole (39.40%), 1,8-cineole (28.00%) and sabinene (8.50%). The plant exhibited antibacterial activity against *S. agalactiae* and *S. aureus* with zones of inhibition of 12.0 and 13.0 mm, respectively. No MIC values and standard were reported [52]. The leaf EO of *O. nonata* was tested against five bacteria strains (*Staphylococcus aureus*, *S*. *epidermidis*, *Enterococcus faecalis* and *E. coli*). Moderate activity was observed against *S*. *aureus* with inhibition zones of 12.0 mm, and against *S*. *epidermidis* and *E. faecalis* with inhibition halos of 10 mm. EO composition, MIC values and standard were not reported [42]. 

The antibacterial potential of *Licaria, Nectandra* and *Ocotea* species was also evaluated by the microdilution method. The essential oil of *O. caudata* revealed bicyclogermacrene (29.60%), germacrene D (19.90%) and β-caryophyllene (9.60%) as major constituents. *O. cujumary* was mainly composed of β-caryophyllene (22.20%), caryophyllene oxide (12.40%) and 2-tridecanone (7.30%). Conversely, *O. caniculata* was dominated by β-selinene (20.30%), β-caryophyllene (18.90%) and 7-*epi*-α-selinene (14.30%). These species were collected in Caxiuanã National Forest (Melgaço, PA) and their antibacterial activity was evaluated against *Bacillus cereus*, *E. coli*, *P. aeruginosa*, *S. aureus* and *Staphylococcus epidermidis*. The antibiotic Gentamicin was used as positive control [36]. 

These three *Ocotea* EOs showed strong activity against *E. coli* (MIC 19.50 μg/mL) and weak potential against *S. aureus* (MIC 625 μg/mL). *O. cujumary* exhibited moderate activity against *B. cereus* (MIC 312.50 μg/mL) and *S. epidermidis* (MIC 312.50 μg/mL). *O. caudata* showed moderate (MIC 312.50 μg/mL) and weak potential (625.00 μg/mL), respectively. The species *O. caniculata* exhibited weak activity against *B. cereus* and *S. epidermidis* (MIC 625.0 μg/mL) [36]. In addition, some EO components such as α-pinene, β-pinene, limonene, β-caryophyllene, α-humulene and germacrene D indicated antimicrobial activity (MIC 156.0–625.0 μg/mL) against *E. coli*, *S. epidermidis*, *B. cereus* and *S. aureus*. The compound caryophyllene oxide was only active against *B. cereus* (MIC 156.0 μg/mL) [36].

Three profiles of *L. rigida* from the Caxiuanã National Forest (Melgaço, PA) had their EO composition evaluated. Profile I had 6-methoxy-elemicin as the major component from leaves (51.86%), twigs (63.31%) and branches (39.55%). Profile II had β-caryophyllene (76.09%) in the leaves, and caryophyllene oxide (29.88%), 14-hydroxy-9-*epi*-β-caryophyllene (10.28%) and β-caryophyllene (8.92%) in the twigs. Profile 3 was rich in δ-cadinene (10.53%), β-caryophyllene (9.73%) and β-bourbonene (9.44%) in the leaves, and in δ-cadinene (12.04%), terpinen-4-ol (10.67%) and selin-11-en-4α-ol in the branches (7.67%).

Three profiles of the leaves of *L. rigida* from the Caxiuanã National Forest (Melgaço, PA) had their EO composition evaluated. In the leaves, the main compounds were: β-caryophyllene (76.09%), α-humulene (6.61%), and viridiflorene (4.65%) (profile I); δ-cadinene (10.53%), β-caryophyllene (9.73%) and β-bourbonene (9.44%) (profile II); 6-methoxy-elemicin (51.86%), caryophyllene (15.33%), and selin-11-en-4α-ol (9.68%). The oils from twigs and branches of these specimens displayed two profiles. The most abundant compounds in the twigs were 14-hydroxy-9-*epi*-β-caryophyllene (10.28%) and β-caryophyllene (8.92%) (profile I) and 6-methoxy-elemicin (63.31%) and selin-11-en-4α-ol (23.99%) (profile II). δ-cadinene (12.04%), terpinen-4-ol (10.67%) and selin-11-en-4α-ol (7.67%) (profile I) and 6-methoxy-elemicin (39.55%) and selin-11-en-4α-ol (21.82%) (profile II) in the branches. All EOs indicated strong activity against *E. coli* (MIC <19.5 μg/mL). The antibiotic Gentamicin was applied as the reference standard [27]. Leaves of *N. megapotamica* collected in Cananeia (SP, Brazil) showed potential against *S. aureus* (71.0%) and *P. aeruginosa* (51.0%) at a concentration of 3.125 µL/mL. The antibiotics chloramphenicol, amikacin and nystatin were used as positive controls. However, the EO composition and the MIC values were not reported [61]. 

The leaf EO of *N. puberula* from Santarém (PA) was rich in apiole (22.20%), β-caryophyllene (15.10%) and β-pinene (13.30%). In contrast, *N. cuspidata* from Caxiuanã National Forest (Melgaço, PA) was dominated by β-caryophyllene (26.90%), bicyclogermacrene (16.0%) and spathulenol (5.20%). Both specimens exhibited activity against *Escherichia coli* (MIC 19.50 μg/mL), *Bacillus cereus* (MIC 312.50–625.0 μg/mL), *Staphylococcus aureus* (MIC 312.50–625.0 μg/mL), and *Staphylococcus epidermidis* (MIC 625.0 μg/mL). The antibiotic Gentamicin was employed as the reference standard [29].

The leaf EOs of three *Nectandra* species from Botocatu (SP) had their antibacterial activity evaluated seasonally by the resazurin-based assay with 96-well plates. In the winter, spring and fall*, N. megapotamica* was mainly composed of α-pinene (20.10%, 18.20%, 25.10%), β-pinene (18.50, 16.20%, 22.30%) and bicyclogermacrene (10.60%, 8.70%, 9.10%). In the summer, bicyclogermacrene (14.80%), α-phellandrene (11.0%), and α-pinene (10.10%) were its major constituents [23]. The oils exhibited as inactive against *Staphylococcus aureus* (winter, MIC 1.05%; spring, MIC 1.90%; summer, MIC 1.90%; fall, MIC 3.0%) and *Escherichia coli* (winter, MIC 2.25%; spring, MIC 5.50%; summer, MIC 6.50%; fall, MIC 6.75%). The positive control applied was 0.01% resazurin [23].

The leaf EO of *N. lanceolata* was dominated by: (1) *iso*-bicyclogermacrenal (30.0%), spathulenol (20.20%), and rosadiene (6.10%) in the winter; (2) *iso*-bicyclogermacrenal (34.10%) bicyclogermacrene (12.60%), and spathulenol (7.60%) in the spring; (3) *iso*-bicyclogermacrenal (34.30%), spathulenol (15.90%), and bicyclogermacrene (4.80%) in the summer; and (4) *iso*-bicyclogermacrenal (41.80%), spathulenol (11.90%), and rosadiene (3.60%) in the fall. These EOs also showed limited potential against *Escherichia coli* (winter, MIC 7.50%; spring, MIC 4.0%; summer, MIC 10.10%; fall, MIC 10.10%), and *Staphylococcus aureus* (winter, MIC 0.60%; spring, MIC 0.70%; summer, MIC 0.55%; fall, MIC 0.55%) [23].

The species *N. grandiflora* had in the spring, summer, fall and winter *iso*-bicyclogermacrenal (39.1%, 27.8%, 39.6%, 29.6%), spathulenol (13.3%, 18.5%, 11.1%, 20.10%), and rosadiene (11.6%, 16.6%, 11.2%, 15.1%) as major compounds. The plant exhibited very weak antibacterial properties against *Escherichia coli* (winter, MIC 6.5%; spring, MIC 4.25%; summer, MIC 10.1%; Fall, MIC 10.1%), and S*taphylococcus aureus* (winter, MIC 1.9%; spring, MIC 1.8%; summer, MIC 1.9%; fall, MIC 3.0%) throughout the seasons [23].

### 6.2. Antifungal Activity

The leaf EO of *N. lanceolata* was mainly composed of β-caryophyllene (32.50%), bicyclogermacrene (27.80%) and spathulenol (11.80%). On the other hand, *N. megapotamica* was represented by bicyclogermacrene (33.40%), germacrene D (16.80%) and limonene (14.10%). Both species were collected in Barracão (RS) and had moderate activity against the dermatophytes *Trichophyton rubrum, T. mentagrophytes*, *Microsporum canis* and *M. gypseum* (MIC 250–500µg/mL). The assays were performed by the microdilution method, and terbinafine was applied as reference standard (MIC 0.004–0.016µg/mL). In addition, a combination of each oil with ciclopirox was evaluated regards its synergistic effect. The interaction was defined quantitatively as a fractional inhibitory concentration (FIC). The synergism was indicated when FIC values were below 0.5. The results indicated that the *N. lanceolata* EO with ciclopirox had a synergistic effect (FICI 0.375) for *T. rubrum* (TRU43) and *M. canis* (MCA29), which means that the concentration of the active antifungal agent can be reduced when in combination with the EO [31].

The oil of *O. lancifolia* from Santa Maria (RS) was evaluated against the phytopathogenic fungus *Fusarium moniliforme* in different seasons. In April, the leaf EOs were mainly composed of caryophyllene oxide (40.61%), *allo*-himachalol (6.51%) and bicyclogermacrene (6.75%) where the highest mycelial inhibition was found (67.50%) at 1.0 µL/mL. Inflorescences and fruits were collected in April and September, respectively. Inflorescences had caryophyllene oxide (34.90%), bicyclogermacrene (8.10%) and β-chymopodiol (6.0%) as major constituents while fruits were dominated by caryophyllene oxide (52.10%) and bicyclogermacrene (9.90%). The percentage of mycelial growth inhibition varied from 63.0–65.0% at 1.0 µL/mL, and nystatin was used as positive control. All EOs showed higher antifungal activity than nystatin (30.0%), but no MIC values were reported [50].

Different concentrations of EOs of leaves of *N. grandiflora* from Jaguari (RS) were tested on the growth of *Pycnoporus sanguineus* (white-rot fungus) and *Gloeophyllum trabeum* (brown-rot fungus). The oil was dominated by dehydrofukinone (26.85%), valencene (6.89%) and kaurene (6.03%). The oil exhibited a LC_50_ (Lethal concentration is the amount of the oil required to kills 50% of the larvae) of 0.39 μL/mL against the fungus *G. trabeum* and a LC_50_ of 1.22 μL/mL against *P. sanguineus.* The bioactivity can be explained by the presence of the major compound dehydrofukinone. In a parallel experiment, this compound was isolated and had its antifungal activity evaluated. It showed mycelial inhibition ranging from 76.06% and 79.45% in comparison to the pure EO with 80.56%. The assay was performed by the radial growth technique, but no reference standard was reported [45].

The antifungal effect of leaves of *Ocotea* species from Borba (AM) was also evaluated by the disc diffusion method. *L. puchury-major* showed strong inhibitory effect against some fungi species frequently found in hospitals and potentially responsible for opportunistic mycoses such as *Rhodotorula* spp., *Candida albicans*, *Fusarium* spp., *Alternaria* spp. and mixed molds with zones of inhibition varying from 31.0 to 37.30 mm. The highest effect was found for *Aspergillus fumigatus* with a halo of 64.30 mm diameter. The EO composition and MIC values were not reported. The authors used 6-mm sterile paper disks containing 15 µL of each EO. Zones of inhibition ≥20 mm were considered strongly inhibitory [62]. A different specimen of *L. puchury-major* had its activity evaluated. The EO was mainly composed of safrole (39.40%), 1,8-cineole (28.0%) and sabinene (8.50%). Pure oil indicated strong antifungal potential (29.0 and 40.0 mm) against two yeast species (*Rhodotorula* sp. and *Candida* sp.) and a mixture of molds. A paper disc without oil was used as negative control. MIC values and reference standards were not mentioned in the manuscript [52]. 

### 6.3. Cardiovascular Activity

EOs of *O. duckei* from Santa Rita (PB) had their cardiovascular activity evaluated in 52 normotensive mice at 1.0, 5.0, 10.0 and 15.0 mg/kg. Leaves were rich in β-caryophyllene (60.54%), α-humulene (4.63%), and δ-selinene (4.40%). EOs in the tested concentrations induced hypotension (7.0%, 15.0%, 21.0% and 37.0%, respectively) followed by bradycardia (3.0%, 9.0%, 18.0% and 53.0%, respectively). Additionally, stem barks were dominated by β-eudesmol (27.51%), α-pinene (9.02%), and limonene (6.65%). The oil induced hypotension (8.0%, 25.0%, 38.0%, 27.0%) followed by bradycardia (5.0%, 22.0%, 53.0%, 49.0%). Fruits had limonene (30.12%), β-pinene (12.25%), and α-pinene (9.89%), while roots were mainly constituted of elemol (24.31%), β-eudesmol (13.44%), and β-elemene (16.69%). EOs from fruits induced hypotension (6.0%, 8.0%, 18.0% and 26.0%) followed by bradycardia (3.0%, 3.0%, 12.0% and 35.0%). Roots also induced hypotension (4.0%, 20.0%, 33.0%, 25.0%) and bradycardia (3.0%, 30.0%, 57.0% and 35.0%) at 1.0%, 5.0%, 10.0% and 15.0 mg/kg [38]. 

### 6.4. Reduction of Motor and Anesthetic Activity

The leaf EO of *O. acutifolia* from São Francisco de Assis (RS) was mainly composed of caryophyllene oxide (56.90%), calarene epoxide (11.74%), and τ-elemene (8.17%). Anesthesia induction and recovery was evaluated in silver catfish (*Rhamdia quelen*) in six stages: light and deep sedation, partial and total loss of equilibrium, deep anesthesia and medullar collapse. Anesthesia was reached with 300–900 µL/L (13–18 min) of oil, and recovery time was greater than 30 min. In addition, blood glucose levels were evaluated since they are a common indicator of stress response. The EO of *O. acutifolia* (150 µL/L) promoted an increase in blood glucose level. The long induction and recovery times can likely be attributed to the hydrophobic characteristics of the EO [49]. 

EOs of young and old leaves of *N. megapotamica* from Santa Maria (RS) were dominated by bicyclogermacrene (46.5%; 34.6%), α-pinene (26.8%; 26.2%), and β-pinene (7.9/12.3%). Its anesthetic potential was studied in the fish species *Centropomus parallelus.* Both EOs (young and old) were efficient, inducing mild sedation at 30 μL/L (1.3–3.2 min) and deep anesthesia at 150 μL/L (5.6–8.0 min). However, the oils were not able to prevent the stress of anesthesia and transport which was indicated by the elevated glucose and lactate plasma levels [32]. Furthermore, seeds of *L. puchury-major* were dominated by safrole (51.3%), 1,8-cineole (25.50%) and eugenol (3.30%). The oil reduced motor activity in rats at 50–100 mg/kg and anesthetized mice at 800 mg/kg for more than 1 h. The EO at 200 mg/kg also protected the animals against transcorneal electroshock [16]. However, no standard compound was reported for either study. 

### 6.5. Antioxidant Activity 

The antioxidant potential of *Licaria*, *Nectandra* and *Ocotea* species was evaluated by the 2,2-diphenyl-1-picryhydrazyl (DPPH) radical-inhibitory assay. The leaf EO of *L. rigida* from Melgaço (PA) was rich in 6-methoxy-elemicin (51.86%), β-caryophyllene (15.33%) and selin-11-en-4α-ol (9.68%). The antioxidant potential of its EO at a concentration of 2.50 mg/mL was 718.10 ± 106.50 mg TE/mL. The bioactivity was expressed as milligrams of Trolox (standard) equivalent per milliliter of the sample [27]. Leaves of *L. martiniana,* collected in Belém (PA), had β-caryophyllene (41.70%), β-selinene (7.90%) and linalyl isovalerate (5.90%) as major constituents. Stems were mainly composed of linalool (6.50%), β-caryophyllene (21.40%) and spathulenol (11.5%). The EO from the leaves and stems showed IC_50_ >1000 μg/mL in comparison to quercetin at 3.13 μg/mL (IC_50_, the concentration of an inhibitor to promote 50% of reduction of DPPH radicals) [25].

The oil from the leaves of *N. megapotamica* from Barracão (RS) had bicyclogermacrene (33.40%), germacrene D (16.80%) and limonene (14.10%) while *N. lanceolata* had β-caryophyllene (32.5%), bicyclogermacrene (27.80%) and spathulenol (11.80%) as major compounds. *N. lanceolata* oil at 250.0 µg/mL indicated antioxidant activity above 50% while *N. megapotamica* showed free-radical inhibition of around 42.0% in comparison to rutin, the reference standard [31]. 

The species *O. odorifera,* containing safrole (40.23%), camphor (34.35%) and limonene (7.42%), showed 33.96% and 86.45% of free radical inhibition at 10.0 and 150.0 μg/mL [54]. The leaf EO of *O. bicolor* from Curitiba (PA) was mainly composed of δ-cadinene (7.39%), β-sesquiphellandrene (6.67%) and β-elemene (5.41%). The specimen exhibited weak antioxidant activity with IC_50_ >500 μg/mL in comparison to the reference standards of ascorbic acid (102.5%) and rutin (29.21%) [34]. 

### 6.6. Cytotoxic Activity 

Essential oils of *Licaria*, *Nectandra* and *Ocotea* species were evaluated regarding their cytotoxic potential by the MTT method. *L. rígida* (sample LR1) from Caxiuanã National Forest, Melgaço (PA), had 6-methoxy-elemicin (63.31%), selin-11-en-4α-ol (23.99%) and α-selinene (2.45%) as the major components of the branch EO. The specimen showed bioactivity against human mammary adenocarcinoma cell line MCF-7 with IC_50_ 95.1 μg/mL. The leaf EO of sample LR3 also exhibited anticancer potential with IC_50_ 66.5 μg/mL. Its major compounds were δ-cadinene (10.53%), β-caryophyllene (9.73%) and β-bourbonene (9.44%). Tingenone with IC_50_ of 16.8 μg/mL was used as the reference drug [27].

Furthermore, *N. puberula* from Santarém (PA) had apiole (22.20%), β-caryophyllene (15.10%) and β-pinene (13.30%) in its leaves while *N. cuspidata* from Caxiuanã National Forest, Marajó Island (PA), had β-caryophyllene (26.90%), bicyclogermacrene (16.0%) and spathulenol (5.20%). The cytotoxic activities of *N. puberula* and *N. cuspidata* were evaluated against MCF-7 cells (Michigan Cancer Foundation-7), and the IC_50_ values were 64.5 and 117.10 μg/mL, respectively [29]. Leaves of *N. leucantha* from Cubatão (SP), containing bicyclogermacrene (28.44%), germacrene A (7.34%) and α-pinene (6.59%), displayed significant cytotoxic activity against the murine melanoma subline (B16F10-Nex2) with IC_50_ 33.0 μg/mL, human glioblastoma (U-87) with IC_50_ of 75.95 μg/mL and human cervical carcinoma (HeLa) with IC_50_ 60.0 μg/mL [33]. Specimens from the genus *Ocotea* collected in the Caxiuanã National Forest, Melgaço (PA), also had their leaf EO cytotoxic potentials evaluated on MCF-7 cells. *O. caudata* was mainly composed of bicyclogermacrene (29.60%), germacrene D (19.90%) and β-caryophyllene (9.60%), and had IC_50_ 64.0 μg/mL. *O. cujumary* had β-caryophyllene (22.20%), caryophyllene oxide (12.40%) and 2-tridecanone (7.30%) as major compounds and showed IC_50_ 63.90 μg/mL. *O. caniculata* was mainly composed of β-selinene (20.30%), β-caryophyllene (18.90%) and 7-*epi*-α-selinene (14.30%) and had IC_50_ 67.70 μg/mL [36].

### 6.7. Toxicological Activity 

The species *L. canella* from Manaus (AM) showed benzyl benzoate (69.70%), α-pinene (3.54%) and α-copaene (4.99%) as its leaf EO major components. Its toxicological activity was evaluated through the MTT method against mice peritoneal macrophages. The oil showed low toxicity with IC_50_ 6.20 µg/mL in comparison to the standard pentamidine (IC_50_ 24.40 µg/mL). Its EO toxicity was also evaluated by the brine shrimp (*Artemia salina*) lethality test where DMSO was used as negative (LC_50_ >1000 µg/mL) and lapachol as positive control (LC_50_ 23.0 µg/mL). The results indicated high toxicity with LC_50_ 5.25 µg/mL [56]. 

EOs of *Nectandra* species had their toxicological effect evaluated by the sulforhodamine B assay on sarcomas (J774A.1) and fibroblast (NIH/3T3) cells. *N. megapotamica* (sample 1) from Campo Grande (MS) had elemicin (41.70%), (*E*)-asarone (19.70%) and α-pinene (8.50%) as its stem bark major constituents. NIH/3T3 cells treated with EO showed IC_50_ 162.30 µg/mL while J774A.1 exhibited IC_50_ 221.60 µg/mL. In contrast, the stem bark of *N. megapotamica* (sample 2) was dominated by (*E*)-asarone (42.40%), α-cadinol (14.40%) and δ-cadinene (5.80%). Fibroblast cells lines NIH/3T3 indicated IC_50_ 252.60 µg/mL, and J774A.1 sarcoma cells showed IC_50_ 415.60 µg/mL [28].

In addition, the stem bark EO of *N. gardneri* from Campo Grande (MS) was mainly composed of intermedeol (58.20%), α-amorphene (8.0%) and agarospirol (4.0%). NIH/3T3 cells indicated IC_50_ 51.60 µg/mL while J774A.1 cell line showed IC_50_ 29.9 µg/mL. Leaves of *N. hihua* from Maracaju (MS) were mainly composed of bicyclogermacrene (28.1%), germacrene D (13.80%) and β-caryophyllene (9%). In this case, NIH/3T3 cell lines treated with the oil showed IC_50_ 54.90 µg/mL while J774A.1 exhibited IC_50_ 29.80 µg/mL. The leaf EO of *N. amazonum* collected in Cáceres (MS) was mainly constituted of β-caryophyllene (28.50%), germacrene B (14.80%), intermedeol (16.20%). Fibroblast cell lines NIH/3T3 and J774A.1 sarcoma cells exhibited IC_50_ 58.0 µg/mL and 29.40 µg/mL. Overall, the oils showed low toxicity on mammalian cells in comparison to the positive control amphotericin B with IC_50_ 2.20 and 4.30 µg/mL, respectively [28]. 

Leaves of *O. odorifera* from Machado (MG) were mainly constituted of safrole (36.30%), γ-cadinene (6.60%) and camphor (6.50%). The EO toxicological effect was evaluated in peritoneal macrophages of BALB/c mice and exhibited CC_50_ 49.52 μg/mL in comparison to the positive control amphotericin B with CC_50_ 51.86 μg/mL [55]. The leaf EOs of some *Ocotea* species were also tested by the brine shrimp lethality assay. For instance, *O. bicolor* from Curitiba (PR), containing δ-cadinene (7.39%), β-sesquiphellandrene (6.67%) and β-elemene (5.41%), showed LC_50_ 40.10 μg/mL in comparison to the positive control prepared with saline solution and sodium dodecylsulfate (SDS) [34]. Additionally, the species *O. notata* from Carapebus (RJ), mainly composed of germacrene A (22.70%), β-caryophyllene (22.90%) and α-pinene (8.70%), exhibited high toxicity with LC_50_ 2.37 μg/mL [42]. 

### 6.8. Leishmanicidal Activity

EO from the leaves of *L. canella* from Manaus (AM), dominated by benzyl benzoate (69.70%), α-pinene (3.54%) and α-copaene (4.99%), inhibited promastigotes of *Leishmania amazonensis,* the etiological agent of leishmaniasis, with IC_50_ 19.0 µg/mL in comparison to pentamidine with IC_50_ 4.80 µg/mL [56]. Similarly, *O. odorifera* from Machado (MG), containing safrole (36.30%), γ-cadinene (6.60%) and camphor (6.50%), exhibited potential against *L. amazonensis* with IC_50_ 4.67 μg/mL in comparison to the standard amphotericin B with IC_50_ 1.88 μg/mL [55].

The antileishmanial activity of *Nectandra* species was studied in peritoneal macrophages infected with the protozoan. The results pointed out that leaf EO of *N. amazonum* from Cáceres (MS) inhibited the amastigote form of *L. infantum* (IC_50_ 31.1 µg/mL)*,* the etiological agent of visceral leishmaniasis, and *L. amazonensis* (IC_50_ 22.1 µg/mL). The oil had β-caryophyllene (28.50%), germacrene B (14.80%) and intermedeol (16.20%) as major compounds. In addition, stem bark EO of *N. gardneri* from Campo Grande (MS), rich in intermediol (58.20%), α-amorphene (8.0%) and agarospirol (4.0%), inhibited amastigotes of *L. infantum* and *L. amazonensis* with IC_50_ 2.70 and 2.10 µg/mL, respectively. The leaf EO of *N. hihua* from Maracaju (MS) was active against *L. infantum* amastigotes with IC_50_ 2.70 µg/mL and *L. amazonensis* amastigotes with IC_50_ 2.10 µg/mL in comparison to reference drug amphotericin B with IC_50_ 0.3 and 0.2 µg/mL, respectively. Its essential oil was rich in bicyclogermacrene (28.1%), germacrene D (13.8%) and β-caryophyllene (9.0%) [28].

Stem bark of *N. megapotamica* (sample 1) from Campo Grande (MS) had elemicin (41.70%), (*E*)-asarone (19.70%) and α-pinene (8.50%) as major constituents. The oil showed activity against *L. amazonensis* with IC_50_ 19.0 µg/mL. Similarly, stem bark of *N. megapotamica* (sample 2), containing (*E*)-asarone (42.40%), α-cadinol (14.40%) and δ-cadinene (5.80%), showed potential against *L. infantum* and *L. amazonensis* amastigotes with IC_50_ 12.50 and 21.30 µg/mL, respectively. In this study, amphotericin B was employed as a positive control against both *L. infantum* (IC_50_ 0.3 µg/mL) and *L. amazonensis* (IC_50_ 0.20 µg/mL) [28].

### 6.9. Antichemotactic Activity

Chemotaxis, the migration and accumulation of inflammatory cells in the site of injury or infection, corresponds to the principal stage of the inflammatory process (Medzhitov, 2008). For this reason, the potential to inhibit leukocyte migration was evaluated in *N. lanceolata* and *N. megapotamica* leaf essential oil from Barracão (RS) by the Boyden chamber method. The positive control indomethacin inhibited the migration by 62.9% at 10.0 µg/mL. *N. lanceolata,* rich in β-caryophyllene (32.50%), bicyclogermacrene (27.80%) and spathulenol (11.80%), showed inhibition of 30.70–96.70% in leukocytes treated with concentrations varying from 0.625 µg/mL to 10.0 µg/mL. *N. megapotamica*, dominated by bicyclogermacrene (33.40%), germacrene D (16.80%) and limonene (14.10%), exhibited similar results (34.5–94.1%) in comparison to the negative control with neutrophils solution without antichemotactic agent [31]. The species *N. megapotamica* collected in Cananéia (SP) exhibited anti-inflammatory potential by the same method. However, no chemical composition or inhibition percentages were reported by the authors [61]. 

### 6.10. Other Activities

The acaricidal potential of an *Ocotea* species was evaluated against the mite *Tetranychus urticae* Koch. The species *O. gardneri* from Igarassu (PE) was mainly constituted of germacrene D (26.96%), bicyclogermacrene (20.73%) and viridiflorol (5.52%). The leaf EO was tested in concentrations ranging from 1.50 to 2.50 µL/cm^2^, showing percentages of repellency varying from 17.32% to 68% [40]. 

Besides this, *Ocotea* species also showed molluscidal activity. Stem barks of *O. bracteosa* from Santa Rita (PB) had δ-cadinene (12.40%), ledene (11.10%) and globulol (10.10%) as major compounds. The species showed potential against the aquatic mollusk *Biomphalaria glabrata*, the main intermediate host of schistosomiasis in South America, with LC_90_ 8.30 µg/mL. Two control sets were used; one with cupric carbonate at 50 ppm and the other with 0.10% DMSO dechlorinated water [35]. *O. gardneri* containing β-caryophyllene (29.28%), α-pinene (15.40%) and kaurene (18.35%), exhibited molluscicidal activity against *B. glabrata* with LC_90_ 16.50, LC_50_ 9.70 and LC_10_ 2.80 mg/mL, but no controls were indicated [40]. 

The species *O. odorifera* from Marcelino Ramos (RS), dominated by camphor (43.0%), safrole (42.0%) and camphene (6.0%), showed insecticidal effect against *Sitophilus zeamais*, the maize weevil, with LD_50_ 14.10 μL or 0.09 μL/cm^2^ and 100% of mortality after 72 h. Similarly, the repellency bioassay simulating small bins showed repellent effects varying from 0.64 (0.36 μL/cm^3^) to 0.94 (2.9 μL/cm^3^). No positive control was reported [53]. Additionaly, leaves and bark of *N. megapotamica* from Santa Maria (RS) were tested against Coenagrionidae (damselfly) larvae. Larval mortality was evaluated using a concentration of 0.1 µL/mL EO at different exposure times (1 min, 40 min, 1 h, 2 h, 4 h, 6 h, 9 h, and 19 h) and leaves showed only 20% and bark 60% of mortality after 19 h. Unfortunately, the EO composition and standard controls were not reported [63].

## 7. Chemical Composition-Geographic Distribution Correlation 

**Figure 5:**
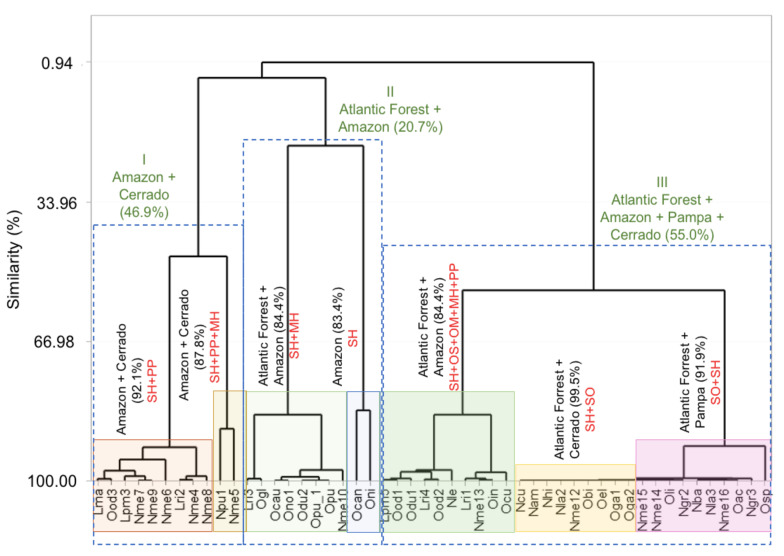
Dendrogram representing the similarity relationship in the oil compositions and geographical occurrence of species of *Licaria*, *Nectandra* and *Ocotea* collected in Brazilian biomes. *Licaria martiniana* (Lma), *Ocotea odorífera* (Ood1, Ood2, Ood3), *L. puchury-major* (Lpm3, Lpm5), *Nectandra megapotamica* (Nme4, Nme5, Nme6, Nme7, Nme8, Nme9, Nme10, Nme12, Nme13, Nme14, Nme15, Nme16), *L. rigida* (Lr1, Lr2, Lr3, Lr4), *N. puberula* (Npu1), *O. glomerata* (Ogl), *O. caudata* (Ocau), *O. notata* (Ono1), *O. duckei* (Odu2), *O. puberula* (Opu_1, Opu), *O. caniculata* (Ocan), *O. nigrescen* (Oni), *O. duckei* (Odu1), *N. lanceolata* (Nle), *O. indecora* (Oin), *O. cujumary* (Ocu), *N. cuspidata* (Ncu), *N. amazonum* (Nam), *N. hihua* (Nhi), *N. lanceolata* (Nla2, Nla3), *Ocotea bicolor* (Obi), *O. elegans* (Oel), *O. gardneri* (Oga1, Oga2), *O. limae* (Oli), *N. grandiflora* (Ngr2, Ngr3), *N. barbellata* (Nba), *O. acutifólia* (Oac), *O. splendens* (Osp).

A multivariate statistical analysis was performed in order to find chemical markers according to geographic occurrence of Lauraceae species. The total percentage of compound classes (monoterpene hydrocarbons (MH), oxygenated monoterpenoids (OM), sesquiterpene hydrocarbons (SH), oxygenated sesquiterpenoids (OS) and phenylpropanoids (PP) for each of the leaf oils was used as variables. The data matrix was standardized by subtracting the mean from individual value of each compound and then subtracted it by the standard deviation. The values were submitted to Hierarchical Cluster Analysis (HCA) the Euclidian distance and complete linkage and absolute correlation coefficient distance were selected as a measure of similarity using the Minitab software (free 390 version, Minitab Inc., State College, PA, USA) (Figure 5).

Based on the dendrogram obtained by HCA, the oils from the leaves of Lauraceae species were classified into three main clusters. Cluster I was composed of 12 samples collected in the biomes Amazon and Cerrado divided into two subgroups, which presented a similarity level of 46.9%. The subgroup I-1, the samples displayed a higher average of sesquiterpene hydrocarbons (52.1%) and phenylpropanoids (29.3%) and a similarity of 92.1%. On the other hand, the oils of subgroup I-2 showed a similarity of 87.8%, and the average of their main compounds were of 39.8%, 30.4%, and 20.9% to sesquiterpene hydrocarbons, phenylpropanoids, and monoterpene hydrocarbons, respectively. Cluster II presented a similarity of 20.7%, and it was composed of 10 samples collected in the biomes Atlantic Forest and Amazon classified into two subgroups. The main classes presented in the subgroup II-1 were sesquiterpene (72.1%) and monoterpene (16.5%) hydrocarbons and only sesquiterpene hydrocarbons (75.8%) to subgroup II-2. These subgroups displayed a similarity level of 84.4% and 83.4%, respectively. 

Cluster II included 29 samples collected in the biomes Atlantic Forest, Amazon, Pampa, and Cerrado with the higher similarity level (55.0%) subdivided into three subgroups. The subgroup III-1 was composed of 10 samples collected in Atlantic Forest and Amazon with a similarity of 84.4%. These oils displayed a higher chemical diversity of the main compounds. The predominant classes were sesquiterpene (35.8%) and monoterpene hydrocarbons (13.0%), oxygenated sesquiterpenoids (21.3%) and monoterpenoids (13.3%), and phenylpropanoids (12.5%). Subgroup III-2 included nine samples rich in sesquiterpene hydrocarbons (57.2%) and oxygenated sesquiterpenoids (35.5%) with a similarity of 99.5% among samples collected in Atlantic Forest and Cerrado. Finally, the subgroup III-3 was formed by ten samples collected in Atlantic Forrest and Pampa biomes and displayed a similarity of 91.9%. These samples displayed a high average of concentrations of oxygenated sesquiterpenoids (47.3%) and sesquiterpene hydrocarbons (36.4%).

In summary, sesquiterpene hydrocarbons were present in all oils extracted from the leaves collected in Brazilian biomes. However, some compound classes were able to discriminate the Lauraceae oils based on their site collection. Samples collected in the Amazon and Cerrado showed high amounts of sesquiterpene hydrocarbons and phenylpropanoids. However, these biomes displayed other chemical profiles. Chemical markers of the Pampa biome were oxygenated sesquiterpenoids followed by sesquiterpene hydrocarbons. Samples from the Amazon and Atlantic Forest showed high contents of sesquiterpene and monoterpene hydrocarbons.

## 8. Conclusions

The genera *Licaria*, *Nectandra*, and *Ocotea* have shown high biodiversity in the territorial extension of Brazil, corresponding about 50% of the Lauraceae species in the country. However, studies focused on their essential oils (EOs) represent only 15% of the total species. According to our bibliographic research, species from the *Licaria* genus were collected only in the Amazon biome, and the Cerrado biome displayed the exclusive occurrence of *Nectandra* species. The essential oils displayed a broad chemical diversity with generally higher amounts of sesquiterpenes, as well as considerable contents of phenylpropanoids, and monoterpenes. Sesquiterpenes were present in all oils extracted from the leaves and its combination with other compound classes could discriminate some chemical markers to species collected, especially from Amazon, Cerrado and Pampa biomes. Various species showed the occurrence of two or more chemical profiles according to its site collection or seasonality, and the EO of *Nectandra megatopomica* was the most studied. The EOs displayed several biological activities, especially as cytotoxic and antimicrobial agents against fungi and bacteria. The results of this review suggest the high economic potential of these essential oils as new agents in the pharmaceutical, cosmetic, and food industries. 

## Figures and Tables

**Table 1:** Essential oil compositions of *Ocotea, Nectandra* and *Licaria* species from Brazil.

Species	Collection Site	Date	Plant Part	Extraction Type	Major Components	References
*L. canella*	Manaus, AM	October, 2007	Leaf	HD	Profile I, dry season: benzyl benzoate (69.70%), α-copaene (4.99%), and α-phellandrene (4.20%)	[56]
*L. canella*	Manaus, AM	February, 2008	Leaf	HD	Profile I, rainy season: benzyl benzoate (73.00%), α-copaene (4.51%), and α-phellandrene (3.33%)	[56]
*L. martiniana*	Belém, PA	March, 2008	Leaf	HD	Profile I: β-caryophyllene (41.70%), β-selinene (7.90%), and linalool isovalerate (5.90%)	[25]
*L. martiniana*	Belém, PA	March, 2008	Stem	HD	Profile I: β-caryophyllene (21.40%), spathulenol (11.50%), and linalool (6.50%)	[25]
*L. puchury-major*	Belém, PA	Not reported	Seed	SD	Profile I: safrole (51.30%), 1,8-cineole (25.50%), and α-terpineol (8.60%)	[16]
*L. puchury-major*	Belém, PA	Not reported	Seed	HD	Profile II: safrole (38.80%), 1,8-cineole (21.70%), and limonene (8.27%)	[57]
*L. puchury-major*	Belém, PA	Not reported	Seed	SD	Profile II: safrole (36.11%), 1,8-cineole (21.12%), and limonene (12.20%)	[15]
*L. puchury-major*	Manaus, AM	July, 2002	Seed	HD	Profile III: safrole (58.40%), dodecanoic acid (13.70%), and α-terpineol (8.40%)	[58]
*L. puchury-major*	Borba, AM	June, 2006	Leaf	HD	Profile I: safrole (39.40%), 1,8-cineole (27.60%), and sabinene (8.50%)	[52]
*L. rigida*	Melgaço, PA	Not reported	Leaf	HD	Profile I: β-caryophyllene (59.40%), caryophyllene oxide (12.10%), and α-humulene (7.80%)	[26]
*L. rigida*	Caxiuanã National Forest, Melgaço, PA	Not reported	Leaf	HD	Profile I: β-caryophyllene (76.09%), α-humulene (6.61%), and viridiflorene (4.65%)	[27]
*L. rigida*	Caxiuanã National Forest, Melgaço, PA	Not reported	Leaf	HD	Profile II: δ-cadinene (10.53%), β-caryophyllene (9.73%), β-bourbonene (9.44%), and α-copaene (8.89%)	[27]
*L. rigida*	Caxiuanã National Forest, Melgaço, PA	Not reported	Leaf	HD	Profile III: 6-methoxyelemicin (51.86%), β-caryophyllene (15.33%), and selin-11-en-4α-ol (9.68%)	[27]
*L. rigida*	Caxiuanã National Forest, Melgaço, PA	Not reported	Twig	HD	Profile I: caryophyllene oxide (29.88%), 14-hydroxy-9-*epi*-β-caryophyllene (10.28%), and β-caryophyllene (8.92%)	[27]
*L. rigida*	Caxiuanã National Forest, Melgaço, PA	Not reported	Twig	HD	Profile II: 6-methoxyelemicin (63.31%), selin-11-en-4α-ol (23.99%), α-selinene (2.45%), and terpinen-4-ol (2.31%)	[27]
*L. rigida*	Caxiuanã National Forest, Melgaço, PA	Not reported	Branch	HD	Profile I: γ-cadinene (12.04%), terpinen-4-ol (10.67%), selin-11-en-4α-ol (7.67%), and ledol (6.68%)	[27]
*L. rigida*	Caxiuanã National Forest, Melgaço, PA	Not reported	Branch	HD	Profile II: 6-methoxyelemycin (39.55%), selin-11-en-4α-ol (21.82%), and terpinen-4-ol (9.97%)	[27]
*N. amazonum*	Cáceres, MS	Not reported	Leaf	HD	Profile I: β-caryophyllene (28.50%), intermedeol (16.20%), and germacrene B (14.80%)	[28]
*N. barbellata*	Ribeirão Grande, SP	Not reported	Leaf	HD	Profile I: δ-cadinene (11.42%), β-caryophyllene (9.79%), and α-muurolol (7.56%)	[3]
*N. cuspidata*	Caxiuanã National Forest, Melgaço, PA	Not reported	Leaf	HD	Profile I: β-caryophyllene (26.90%), bicyclogermacrene (16.00%), and spathulenol (5.20%)	[29]
*N. gardneri*	Campo Grande, MS	Not reported	Stem bark	HD	Profile I: intermedeol (58.20%), α-amorphene (8.00%), agarospirol (4.00%), germacrene D (3.50%) and α-elemene (3.50%)	[28]
*N. grandiflora*	Botocatu, SP	Not reported	Leaf	HD	Profile I, spring, summer, fall and winter: *iso*-bicyclogermacrenal (39.10%, 27.80%, 39.60%, 29.60%), spathulenol (13.30%, 18.50%, 11.10%, 20.10%), rosadiene (11.60%, 16.60%, 11.20%, 15.10%)	[23]
*N. grandiflora*	Jaguari, RS	Not reported	Leaf	HD	Profile II: dehydrofukinone (26.85%), valencene (6.89%), kaurene (6.03%), and selin-11-en-4-α-ol (5.34%)	[45]
*N. grandiflora*	Jaguari, RS	October-november, 2013	Leaf	HD	Profile III: dehydrofukinone (24.70%), bicyclogermacrene (5.93%), and kaurene (5.49%)	[46]
*N. hihua*	Maracaju, MS	Not reported	Leaf	HD	Bicyclogermacrene (28.10%), germacrene D (13.80%), and β-caryophyllene (9.0%)	[28]
*N. lanceolata*	Barracão, RS	Not reported	Leaf	HD	Profile I: β-caryophyllene (32.50%), bicyclogermacrene (27.80%), and spathulenol (11.80%)	[31]
*N. lanceolata*	Mundo Novo, MS	February–march, 2013	Leaf	HD	Profile I: bicyclogermacrene (18.20%), spathulenol (16.90%), and β-caryophyllene (12.45%)	[30]
*N. lanceolata*	Mundo Novo, MS	February–march, 2013	Stem bark	HD	Profile I: guaiol (13.2%), cubenol (7.50%),γ-cadinene (7.5%), and α-eudesmol (7.0%)	[30]
*N. lanceolata*	Botocatu, SP	Not reported	Leaf	HD	Profile II, fall (May), winter (August): *iso*-bicyclogermacrenal (41.8%; 30.0%), spathulenol (11.9%; 20.2%), rosadiene (3.1%; 6.1%)	[23]
					Spring (November), summer (February): *iso*-bicyclogermacrenal (34.1%; 34.3%), bicyclogermacrene (12.1%; 4.8%), spathulenol (7.6%; 15.9%)	
*N. leucantha*	Ecological Park of Pereque, Cubatão, SP	December, 2012	Leaf	HD	Profile I: bicyclogermacrene (28.44%), germacrene A (7.34%), α-pinene (6.59%), and spathulenol (5.82%)	[33]
*N. megapotamica*	Santa Maria-RS	November, 2010–September, 2011	Leaf (young)	HD	Profile I, spring, summer, fall and winter: α-pinene (33.23%, 28.3%, 21.46% and 17.46%), β-pinene (17.8%, 15.43%, 13.86% and 10.36%), bicyclogermacrene (15.4%, 32.93%, 26.83% and 23.1%), germacrene D (6.4%, 10.43%, 9.4% and 10.13%)	[24]
*N. megapotamica*	Santa Maria-RS	November, 2010–September, 2011	Leaf (Adult)	HD	Profile I, spring, summer, fall and winter: α-pinene (36.86%, 34.86%, 24.86%, and 15.5%),β-pinene (18.76%, 20.23%, 15.96%, and 10.06%), bicyclogermacrene (17.96%, 25.5%, 22.1%, and 23.6%), germacrene D (3.53%, 6.36%, 7.83%, and 9.8%).	[24]
*N. megapotamica*	Santa Maria, RS	November, 2010	Leaf (young)	HD	Profile I: bicyclogermacrene (46.47%), α-pinene (26.82%), germacrene D (9.61%), and β-pinene (7.95%)	[32]
*N. megapotamica*	Santa Maria, RS	November, 2010	Leaf (adult)	HD	Profile I: bicyclogermacrene (34.56%), α-pinene (26.19%), β-pinene (12.30%), germacrene D (9.2%)	[32]
*N. megapotamica*	Barracão, RS	Not reported	Leaf	HD	Profile II: bicyclogermacrene (33.40%), germacrene D (16.8%), and limonene (14.1%)	[31]
*N. megapotamica*	Maracaju, MS	April, 2014	Leaf	HD	Profile III: bicyclogermacrene (66.7%), germacrene D (18.2%), and elemicin (5.6%)	[48]
*N. megapotamica*	Ponta Porã, RS	April, 2014	Leaf	HD	Profile IV: δ-elemene (32.2%), bicyclogermacrene (28.2%), and α-asarone (10.3%)	[48]
*N. megapotamica*	Ponta Porã, RS	April, 2014	Leaf	HD	Profile IV: δ-elemene (37.9%), bicyclogermacrene (26.3%), and α-asarone (15.0%)	[48]
*N. megapotamica*	Campo Grande, MS	October, 2013	Leaf	HD	Profile V: α-asarone (22.6%), δ-elemene (15.6%), and α-santalene (11.8%)	[48]
*N. megapotamica*	Campo Grande, MS	November, 2013	Leaf	HD	Profile VI: elemicin (35.9%), bicyclogermacrene (24.8%), and δ-3-carene (10.9%)	[48]
*N. megapotamica*	Campo Grande, MS	November, 2013	Leaf	HD	Profile VII: elemicin (52.7%), and bicyclogermacrene (8.9%), and α-pinene (5.7%)	[48]
*N. megapotamica*	São Paulo-SP	February and August, 2007	Leaf	HD	Profile VIII, summer: α-bisabolol (68.55%) and δ-elemene (12.2%).	[47]
					Profile VIII, winter: α-bisabolol (63.55%) and δ-elemene (22.55%).	
*N. megapotamica*	São Paulo-SP	November, 2014	Leaf	HD	Profile IX: α-bisabolol (59.7%), δ-elemene (13.8%), and *iso*-spathulenol (11.3%)	[48]
*N. megapotamica*	São Paulo-SP	November, 2014	Leaf	HD	Profile X: α-bisabolol (84.3%), germacrene D (4.0%), and β-bisabolene (2.5%)	[48]
*N. megapotamica*	São Paulo-SP	November, 2014	Leaf	HD	Profile XI: α-bisabolol (93.7%), β-ocimene (1.5%) and germacrene D (1.4%)	[48]
*N. megapotamica*	São Paulo-SP	November, 2014	Leaf	HD	Profile XII: *iso*-spathulenol (26.8%), δ-elemene (23.8%), and β-bisabolene (13.3%)	[48]
*N. megapotamica*	São Paulo-SP	November, 2014	Leaf	HD	Profile XIII: β-sesquiphellandrene (32.0%), β-bergamotene (19.0%), and α-bisabolol (8.9%)	[48]
*N. megapotamica*	Botocatu, SP	Not reported	Leaf	HD	Profile XIV, spring (November): α-pinene (18.2%), β-pinene (16.2%), α-phellandrene (10.0%)	[23]
					Summer (February): bicyclogermacrene (14.80%), α-phellandrene (11.0%), α-pinene (10.1%), and β-pinene (9.6%)	
					Fall (May): α-pinene (25.1%), β-pinene (22.3%), and bicyclogermacrene (9.1%)	
					Winter (August): α-pinene (20.1%), β-pinene (18.5%), and bicyclogermacrene (10.6%)	
*N. megapotamica*	Campo Grande, MS	Not reported	Stem bark	HD	Profile I elemicin (41.7%), α-asarone (19.7%), and α- pinene (8.5%)	[28]
*N. megapotamica*	Campo Grande, MS	Not reported	Stem bark	HD	Profile II: α-asarone (42.4%), α-cadinol (14.4%), and τ-cadinol (8.1%)	[28]
*N. puberula*	Santarém, PA	Not reported	Leaf	HD	Profile I: apiole (22.2%), β-caryophyllene (15.1%), and β-pinene (13.3%)	[29]
*N. puberula*	Santarém, PA	Not reported	Branch	HD	Profile I: apiole (28.1%), pogostol (19.8%), and guaiol (11.2%)	[29]
*O. acutifolia*	São Francisco de Assis, RS	May, 2011	Leaf	HD	Profile I: caryophyllene oxide (56.9%), calarene epoxide (11.74%), and τ-elemene (8.17%)	[49]
*O. bicolor*	Curitiba, PR	August, 2015	Leaf	HD	Profile I: δ-cadinene (7.39%), β-sesquiphellandrene (6.67%), β-elemene (5.41%), and α-cadinol (5.23%)	[34]
*O. bracteosa*	Santa Rita, PB	May, 2004	Stem bark	HD	Profile I: δ-cadinene (12.4%), ledene (11.1%), and globulol (10.1%)	[35]
*O. caniculata*	Caxiuanã National Forest, Melgaço, PA	Not reported	Leaf	HD	Profile I: β-selinene (20.3%), β-caryophyllene (18.9%), and 7-epi-α-selinene (14.3%)	[36]
*O. caniculata*	Caxiuanã National Forest, Melgaço, PA	Not reported	Branch	HD	Profile I: selin-11-en-4α-ol (20.6%), β-selinene (12.1%), and 7-epi-α-selinene (9.0%)	[36]
*O. caudata*	Caxiuanã National Forest, Melgaço, PA	Not reported	Leaf	HD	Profile I: bicyclogermacrene (29.6%), germacrene D (19.9%), α-pinene (9.8%), and β-pinene (9.7%)	[36]
*O. caudata*	Caxiuanã National Forest, Melgaço, PA	Not reported	Branch	HD	Profile I: δ-cadinene (13.8%), germacrene D (8.9%), β-guaiene (8.3%), and α-muurolol (7.8%)	[36]
*O. cujumary*	Caxiuanã National Forest, Melgaço, PA	Not reported	Leaf	HD	Profile I: β-caryophyllene (22.2%), caryophyllene oxide (12.4%), 2-tridecanone (7.30%), and δ-cadinene (6.6%)	[36]
*O. cujumary*	Caxiuanã National Forest, Melgaço, PA	Not reported	Branch	HD	Profile I: 2-tridecanone (30.0%), limonene (20.5%), and β-caryophyllene (8.1%)	[36]
*O. cymbarum*	Melgaço, PA	Not reported	Stem bark	HD	Profile I: α-selinene (25.8%), δ-cadinene (18.6%), and terpinen-4-ol (9.0%)	[26]
*O. duckei*	Santa Rita, PB	March, 2005	Leaf	SD	Profile I: β-caryophyllene (60.54%), α-humulene (4.63%), and δ-selinene (4.4%)	[38]
*O. duckei*	Santa Rita, PB	March, 2005	Stem bark	SD	Profile I: β-eudesmol (27.51%), α-pinene (9.02%), limonene (6.65%), and borneol (6.18%)	[38]
*O. duckei*	Santa Rita, PB	March, 2005	Fruit	SD	Profile I: limonene (30.12%), β-pinene (12.25%), and α-pinene (9.89%)	[38]
*O. duckei*	Santa Rita, PB	March, 2005	Root	SD	Profile I: elemol (24.31%), β-elemene (16.69%), and β-eudesmol (13.44%)	[38]
*O. duckei*	Senhorzinho Cabral Forest, Camocim of São Félix, PE	September, 2010	Leaf	HD	Profile II: β-caryophyllene (18.1%), valencene (17.6%), and elemol (6.8%)	[37]
*O. elegans*	Restinga de Jurubatiba National Park, Carapebus, RJ	November, 2014–january, 2015	Leaf	HD	Profile I: sesquirosefuran (92.2%)	[51]
*O. gardneri*	Forest of Cruzina, Igarassú, PE	March, 2008	Leaf	HD	Profile I: germacrene D (26.9%), bicyclogermacrene (21.7%), β-caryophyllene (6.1%), and germacrene B (4.9%)	[39]
*O. gardneri*	Igarassú, PE	Not reported	Leaf	HD	Profile I: germacrene D (26.96%), bicyclogermacrene (20.73%) and viridiflorol (5.52%)	[40]
*O. gardneri*	not reported	Not reported	Leaf	HD	Profile I: β-caryophyllene (29.28%), α-pinene (15.4%), kaurene (18.35%), and β-pinene (8.93%)	[41]
*O. glomerata*	Senhorzinho Cabral Forest, Camocim of São Félix, PE	September, 2010	Leaf	HD	Profile I: aromadendrene (17.3%), β-caryophyllene (14.6%), α-pinene (6.90%), and γ-terpinene (6.40%)	[37]
*O. indecora*	Ribeirão Grande, SP	Not reported	Leaf	HD	Profile I: bicyclogermacrene (29.79%), valerianol (15.12%), β-pinene (11.41%), and spathulenol (11.16%)	[3]
*O. lancifolia*	Santa Maria, RS	April, 2013–march, 2014	Leaf	HD	Profile I: Seasonal study: April, June, August: caryophyllene oxide (36.40–40.6%), *allo*-himachalol (6.2–8.0%), bulnesol (6.0–7.10%), and bicyclogermacrene (5.8–6.1%).	[50]
					May: β-chenopodiol (20.9%), kaurene (11.9%), (*Z*)-nerolidyl acetate (9.3%), and caryophyllene oxide (7.0%).	
					July: β-chenopodiol (17.4%), (*Z*)-nerolidyl acetate (8.7%), α-guaiene (5.0%), and (*E*)-β-ocimene (4.9%).	
					September, October: caryophyllene oxide (42.2/46.4%), bicyclogermacrene (6.3/7.3%), *allo*-himachalol (5.7/5.9%), and calarene epoxide (5.5/6.7%).	
					November, January, February, March: caryophyllene oxide (38.6–42.2%), bicyclogermacrene (6.7–7.80%), *allo*-himachalol (5.9–7.4%)	
*O. lancifolia*	Santa Maria, RS	April and May, 2013	Inflorescences	HD	Profile I: seasonal study, April: caryophyllene oxide (34.9%), bicyclogermacrene (8.1%), and β-chenopodiol (6.0%)	[50]
					May: β-chenopodiol (38.7%), α-guaiene (6.0%), and (*Z*)-nerolidyl acetate (4.5%)	
*O. lancifolia*	Santa Maria, RS	July–november, 2013	Fruit	HD	Profile I: seasonal study, July: β-chenopodiol (17.1%), β-ocimene (6.2%), and γ-muurolene (4.7%)	[50]
					August, September: caryophyllene oxide (46.2%, 52.1%), bicyclogermacrene (8.9%, 9.9%), and β-ocimene (2.8%, 3.1%)	
					October: caryophyllene oxide (48.1%), bicyclogermacrene (6.7%), and (*E*)-*iso*-valencenol (3.8%)	
					November: caryophyllene oxide (27.9%), bicyclogermacrene (6.9%), and *allo*-himachalol (6.7%)	
*O. limae*	Igarassú, PE	March, 2008	Leaf	HD	Profile I: spathulenol (13.3%), β-caryophyllene (12.4%), bicyclogermacrene (11.3%), and germacrene D (10.9%)	[39]
*O. longifólia*	Melgaço, PA	Not reported	Stem bark	HD	Profile I: dillapiole (15.2%), δ-cadinene (20.0%), α-cubebene (6.5%), and α-copaene (5.1%)	[26]
*O. nigrescens*	Manaus, AM	March, 2008	Leaf	HD	Profile I: β-caryophyllene (37.9%), β-pinene (6.9%), α-pinene (6.6%), and α-copaene (6.2%)	[44]
*O. notata*	Restinga de Jurubatiba National Park, Carapebus, RJ	November, 2006	Leaf	SD	Profile I: β-caryophyllene (22.9%), germacrene A (22.7%), α-pinene (8.7%), and β-pinene (6.9%)	[42]
*O. odorifera*	Machado, MG	July, 2016	Leaf	HD	Profile I: safrole (36.3%), γ-cadinene (6.6%), camphor (6.5%), and α-copaene (6.0%)	[55]
*O. odorifera*	Marcelino Ramos, RS	Not reported	Leaf	HD	Profile II: camphor (43.0%), safrole (42.0%), camphene (6.0%), limonene (3.0%)	[53]
*O. odorifera*	Marcelino Ramos, RS	Not reported	Leaf	HD	Profile II: safrole (40.23%), camphor (34.35%), limonene (7.42%), and camphene (5.02%)	[54]
*O. puberula*	Curitiba, PR	Not reported	Leaf	HD	Profile I: β-caryophyllene (31.0%), bicyclogermacrene (14.0%), β-elemene (9.7%), and longifolene (8.7%)	[43]
*O. puberula*	Curitiba, PR	Not reported	Branch	HD	Profile I: bicyclogermacrene (31.0%), β-caryophyllene (14.0%), β-pinene (7.9%), and β-elemene (5.3%)	[43]
*O. splendens*	Manaus, AM	March, 2008	Leaf	HD	Profile I: β-caryophyllene (51.0%), caryophyllene oxide (9.9%), and α-humulene (6.2%)	[44]

**Table 2:** Essential oil compositions and biological activities of essential oils from *Ocotea, Nectandra* and *Licaria* species from Brazil.

Lauraceae Species	Collection Site	Plant Part	Major Components	Bioactivities	References
*L. canella*	Manaus, AM	Leaf	Benzyl benzoate (69.70%), α-copaene (4.99%), and α-phellandrene (4.20%)	Anti-leishmanial (*Leishmania amazonensis*, promastigotes*,* IC_50_ 19.0 µg/mL), cytotoxic (mice BALB-c macrophage, IC_50_ 6.20 µg/mL), toxicological (*Artemia salina* lethality, LC_50_: 5.25 μg/mL)	[56]
*L. martiniana*	Belém, PA	Leaf	**L**: β-caryophyllene (41.7%), β-selinene (7.90%), linalyl isovalerate (5.90%), and linalool (5.30%)	Antioxidant (DPPH method, EC_50_ > 1000 μg/mL), and antiplatelet activities (**L**: 4.24%, S: 36.95%)	[25]
			**S**: β-caryophyllene (21.40%), spathulenol (11.50%), and linalool (6.50%)		
*L. puchury-major*	Belém, PA	Seeds	Profile I: safrole (51.30%), 1,8-cineole (25.50%), and α-terpinen-4-ol (8.60%)	Reduced motor activity in rats (50–100 mg/kg) and anesthetized mice (800 mg/kg) for < 1 h.	[16]
*L. puchury-major*	Belém, PA	Seeds	Profile I: safrole (38.80%), 1,8-cineole (21.70%), and limonene (8.27%)	Antioxidant (DPPH method, IC_50_ 27.8 μg/mL), larvicidal (*Aedes aegypti* LC_50_ 98.9 μg/mL; acaricide (*Tetranychus urticae Koch,* LC_50_ 30.8 μg/mL; filter paper disks method, EO at 500 ppm), insecticidal *Cerataphis lataniae,* LC_50_ 13.5 μg/mL, filter paper disks method, EO at 500 ppm)	[57]
*L. puchury Mayor*	Borba, AM	Not reported	Not reported	Antifungal, disc diffusion technique (*Aspergillus fumigatus, Rhodotorula* spp.*, Candida albicans, Fusarium* spp., *Alternaria* spp.), no MIC values	[62]
*L. puchury-major*	Borba, AM	Leaf	Safrole (39.4%),1,8-cineole (27.60%), sabinene (8.50%), and α-terpineol (7.90%)	Antimicrobial (bacteria: *Streptococcus agalactiae, Staphylococcus aureus*; fungi: *Rhodotorula* spp.*, Candida* spp., agar disc diffusion technique), no MIC values	[52]
*L. rigida*	Caxiuanã National Forest, Melgaço, PA	Leaf	Profile I: β-caryophyllene (76.09%), α-humulene (6.61%), and viridiflorene (4.65%) (**L-I**).	Antibacterial (*Escherichia coli*, microbroth dilution method, MIC< 19.50 µg/mL to **L-I**, **L-II**, and **L-III**); Cytotoxic (MCF-7 mammary adenocarcinoma, MTT assay) IC_50_ 66.50 μg/mL (**L-II**), IC_50_ 158.60 μg/mL (**L-III**); Antioxidant (DPPH method, **L-III** 718.1 ± 106.5 mg.ET/mL);	[27]
			Profile II: δ-cadinene (10.53%), β-caryophyllene (9.73%), β-bourbonene (9.44%), and α-copaene (8.89%) (**L-II**)		
			Profile III: 6-methoxy-elemicin (51.86%), β-caryophyllene (15.33%), selin-11-en-4α-ol (9.68%) (**L-III**)		
*L. rigida*	Caxiuanã National Forest, Melgaço,PA	Twig	Profile I: caryophyllene oxide (29.88%), 14-hydroxy-9-epi-β-caryophyllene (10.28%), and β-caryophyllene (8.92%) (**T-I**)	Antibacterial (*Escherichia coli*, MIC < 19.50 µg/mL, microbroth dilution method to T-I, and T-II)	[27]
			Profile II: 6-methoxy-elemicin (63.31%), selin-11-en-4α-ol (23.99%), and α-selinene (2.45%) (**T-II**).		
*L. rigida*	Caxiuanã National Forest, Melgaço, PA	Branch	Profile I: γ-cadinene (12.04%), terpinen-4-ol (10.67%), selin-11-en-4α-ol (7.67%), ledol (6.68%) (**B-I**).	Cytotoxic (MCF-7 mammary adenocarcinoma, MTT assay): IC_50_ 110.70 μg/mL (**B-I**) and IC_50_ 95.10 μg/mL (**B-II**). Antibacterial (*Escherichia coli*, MIC< 19.50 µg/mL, microbroth dilution method)	[27]
			Profile II: 6-methoxy-elemicin (39.55%), selin-11-en-4α-ol (21.82%), and terpinen-4-ol (9.97%) (**B-II**).		
*N. amazonum*	Cáceres, MS	Leaf	β-caryophyllene (28.50%), intermediol (16.20%), and germacrene B (14.80%)	Anti-leishmanial (*Leishmania infantum*, amastigotes, IC_50_ 31.90 µg/mL; *L. amazonensis*, amastigotes, IC_50_ 22.10 µg/mL). Cytotoxic, fibroblast cells (NIH/3T3, IC_50_ 58.0 µg/mL); sarcoma cells (J774.A1, IC_50_ 29.40 µg/mL)	[28]
*N. cuspidata*	Caxiuanã National Forest, Melgaço, PA	Leaf	β-caryophyllene (26.9%), bicyclogermacrene (16.0%) and spathulenol (5.2%)	Antibacterial, (*Escherichia coli*, MIC 19.50 μg/mL; *Bacillus cereus*, MIC 312.50–625.0 μg/mL; *Staphylococcus aureus,* MIC 312.50–625.0 μg/mL; *Staphylococcus epidermidis,* MIC 625.0 μg/mL, microbroth dilution method), cytotoxic, MCF-7 breast tumor cells (IC_50_ 117.10 μg/mL)	[29]
*N. gardneri*	Campo grande, MS	Stem bark	Intermediol (58.20%), α-amorphene (8.0%), agarospirol (4.0%), germacrene D (3.50%), α-elemene (3.50%)	Anti-leishmanial (*Leishmania infantum*, amastigotes, IC_50_ 2.70 µg/mL; *L. amazonensis*, amastigotes, IC_50_ 2.10 µg/mL). Cytotoxic, fibroblast cells (NIH/3T3, IC_50_ 51.60 µg/mL); sarcoma cells (J774A.1, IC_50_ 29.90 µg/mL)	[28]
*N. grandiflora*	Botocatu, SP	Leaf	Profile I, spring, summer, fall and winter: *iso*-bicyclogermacrenal (39.10%, 27.80%, 39.60%, 29.60%), spathulenol (13.30%, 18.50%, 11.10%, 20.10%), rosadiene (11.60%, 16.60%, 11.20%, 15.10%)	Antibacterial, resazurin-based assay: *Escherichia coli* (winter, MIC 6.50%; spring, MIC 4.25%; summer, MIC 10.10%; fall, MIC 10.10%), and S*taphylococcus aureus* (winter, MIC 1.90%; spring, MIC 1.80%; summer, MIC 1.90%; fall, MIC 3.0%)	[23]
*N. grandiflora*	Jaguari, RS	Leaf	Profile II: dehydrofukinone (26.85%), valencene (6.89%), kaurene (6.03%), 4,5-di-*epi*-aristolochene (5.41%)	Antifungal (*Pycnoporus sanguineus,* LC_50_ 1.22 μL/mL; *Gloeophyllum trabeum,* LC_50_ 0.39 μL/mL, radial growth technique)	[45]
*N. grandiflora*	Jaguari, RS	Leaf	Profile III: dehydrofukinone (24.70%), bicyclogermacrene (5.93%), and kaurene (5.49%)	Sustained sedative effect in silver catfish (*Rhamdia quelen*) for 12 h at 10–20 ug/mL	[46]
*N. hihua*	Maracaju, MS	Leaf	Bicyclogermacrene (28.10%), germacrene D (13.80%), β-caryophyllene (9.0%), 9-*epi*-β-caryophylene (7.0%)	Antileishmanial (*Leishmania infantum*, amastigotes, IC_50_ 0.20 µg/mL; *L. amazonenses*, amastigotes, IC_50_ 24.20 µg/mL). Cytotoxic, fibroblast cells (NIH/3T3, IC_50_ 54.90 µg/mL); sarcoma cells (J774A.1, IC_50_ 29.80 µg/mL)	[28]
*N. lanceolata*	Barracão, RS	Leaf	Profile I: β-caryophyllene (32.5%), bicyclogermacrene (27.8%), and spathulenol (11.8%)	Antifungal (*Trichophyton rubrum, Trichophyton mentagrophytes, Microsporum canis* and *Microsporum gypseum,* MIC 250–500 μL/mL, microdilution method); antioxidant, DPPH method (250 µg/mL, above 50% inhibition); antichemotactic effect (leukocyte migration inhibition, 30.70–96.70%)	[31]
*N. lanceolata*	Novo Mundo, MS	Leaf and Bark	Profile II: bicyclogermacrene (18.20%), spathulenol (16.70%), and β-caryophyllene (12.45%).	Cytotoxic (K562 leukemia) TGI = 72.40 and 14.60 mg/mL; U251 glioma, TGI = 75.80 and 37.30 mg/mL.	[30]
			Bark: Guaiol (13.20%), cubenol (7.60%), γ-cadinene (7.60%), α-pinene (6.90%)		
*N. lanceolata*	Botocatu, SP	Leaf	Fall and winter: *iso*-bicyclogermacrenal (41.80/30.0%), spathulenol (11.90/20.20%), rosadiene (3.10/6.10%)	Antibacterial, resazurin-based assay: *Escherichia coli* (winter, MIC 7.50%; spring, MIC 4.0%; summer, MIC 10.10%; fall, MIC 10.10%), and *Staphylococcus aureus* (winter, MIC 0.60%; spring, MIC 0.70%; summer, MIC 0.55%; fall, MIC 0.55%)	[23]
			Spring and summer: *iso-*bicyclogermacrenal (34.10/34.30%), bicyclogermacrene (12.10/4.80%), spathulenol (7.60/15.90%)		
*N. leucantha*	Ecological Park of Pereque, Cubatão, SP	Leaf	Bicyclogermacrene (28.44%), germacrene A (7.34%), and α-pinene (6.59%)	Cytotoxic (B16F10-Nex2 murine melanoma, IC_50_ 33 µg/mL; U87 human glioblastoma, IC_50_ 75.95 µg/mL; HeLa human cervical carcinoma, IC_50_ 60 µg/mL)	[33]
*N. megapotamica*	Cananéia, SP	Leaf	Not reported	Antibacterial (*Escherichia coli,* 20.20%; *Staphylococcus aureus*, 71.0%; *Pseudomonas aeruginosa,* 51.0%, microdilution method); anti-inflamatory, leukocyte migration assay (average distance of 16.20 ± 3.80 mm); cytotoxic (MCF-7 mammary adenocarcinoma, NCI lung great cells carcinoma, KM colon adenocarcinoma, SF glioblastoma, < 50.0%; PC-3 prostate carcinoma, 65.50%; RPMI multiple myeloma, 76.20%). EO at 3.125 µL/mL	[61]
*N. megapotamica*	Santa Maria, RS	Leaf and Bark	Not reported	Larvicidal activity against Coenagrionidae *larvae* (20%, and 60% mortality after 19 h, respectively),EO at 0.1 uL/mL	[63]
*N. megapotamica*	Santa Maria, RS	Leaf (young/old)	Profile I: bicyclogermacrene (46.5/34.6%), α-pinene (26.8/26.2%), β-pinene (7.9/12.3%), and germacrene D (9.6/9.1%)	Anesthetic potential to the fish species *Centropomus parallelus* (mild sedation at 30 μL/L [1.3–3.2 min], and deep anesthesia at 150 μL/L [5.6–8.0 min])	[32]
*N. megapotamica*	Barracão, RS	Leaf	Profile II: Bicyclogermacrene (33.40%), germacrene D (16.80%) and limonene (14.10%)	Antifungal (*Trichophyton rubrum, Trichophyton mentagrophytes, Microsporum canis* and *Microsporum gypseum,* MIC 250–500 μL/mL, microdilution method); antioxidant, DPPH method (250 µg/mL, above 40% inhibition); antichemotactic effect (leukocyte migration inhibition, 34.50–94.10%)	[31]
*N. megapotamica*	Botocatu, SP	Leaf	Profile XIV: spring (November): α-pinene (18.20%), β-pinene (16.20%), α-phellandrene (10.0%), and bicyclogermacrene (8.70%)	Antibacterial, resazurin-based assay: *Escherichia coli* (winter, MIC 2.25%; spring, MIC 5.50%; summer, MIC 6.50%; fall, MIC 6.75%), and *Staphylococcus aureus* (winter, MIC 1.05%; spring, MIC 1.90%; summer, MIC 1.90%; fall, MIC 3.0%)	[23]
			Summer (February): bicyclogermacrene (14.80%), α-phellandrene (11.0%), and α-pinene (10.10%)		
			Fall (May): α-pinene (25.10%), β-pinene (22.30%), and bicyclogermacrene (9.10%)		
			Winter (August): α-pinene (20.10%), β-pinene (18.50%), and bicyclogermanrene (10.60%)		
*N. megapotamica*	Campo grande, MS	Stem bark	Profile I: Elemicin (41.70%), (*E*)-asarone (19.70%), α-pinene (8.50%), (*Z*)-β-ocimene (4.0%)	Antileishmanial (*L. amazonensis*, amastigotes, IC_50_ 19.0 µg/mL), cytotoxic, fibroblast cells (NIH/3T3, IC_50_ 162.30 µg/mL) sarcoma cells (J774A.1, IC_50_ 221.60 µg/mL)	[28]
*N. megapotamica*	Campo grande, MS	Stem bark	Profile II: α-asarone (42.4%), α-cadinol (14.4%), τ-cadinol (8.10%), and δ-Cadinene (5.8%)	Antileishmanial (*Leishmania infantum*, amastigotes, IC_50_ 12.50 µg/mL; *L. amazonenses*, amastigotes, IC_50_ 21.30 µg/mL). cytotoxic, cells fibroblast cells (NIH/3T3, IC_50_ 252.60 µg/mL); sarcoma cells (J774.A1, IC_50_ 415.60 µg/mL)	[28]
*N. puberula*	Santarém, PA	Leaf	Apiole (22.20%), β-caryophyllene (15.10%) and β-pinene (13.30%)	Antibacterial (*Escherichia coli*, MIC 19.50 μL/mL; *Bacillus cereus*, MIC 625.0 μL/mL; *Staphylococcus aureus,* MIC 625.0 μL/mL; *Staphylococcus epidermidis,* MIC 625.0 μL/mL, microbroth dilution method), cytotoxic (MCF-7 mammary adenocarcinoma, IC_50_ 64.5 μg/mL)	[29]
*O. acutifolia*	São Francisco de Assis, RS	Leaf	Caryophyllene oxide (56.90%), calarene epoxide (11.74%), τ-elemene (8.17%),	Anesthetic effect (silver catfish, *Rhamdia quelen*) at 300–900 μL/L (13–18 min).	[49]
*O. bicolor*	Curitiba, PR	Leaf	δ-Cadinene (7.39%), β-sesquiphellandrene (6.67%), β-elemene (5.41%), and α-cadinol (5.23%)	Antioxidant (DPPH method, EC_50_ > 500 μg/mL); antibacterial, microdilution method (*Escherichia coli, Pseudomonas aeruginosa*, *Staphylococcus aureus*, *Enterobacter aerogenes, Klebsiella pneumoniae, Staphylococcus epidermidis* and *Salmonella typhimurium,* MIC > 1000 μg/mL), toxicological (*Artemia salina*, LC_50_ 40.10 μg/mL)	[34]
*O. bracteosa*	Santa Rita, PB	Stem bark	δ-Cadinene (12.40%), ledene (11.10%), globulol (10.1%), and aromadendrene (4.2%)	Molluscicidal (*Biomphalaria glabrata*, LC_90_ 8.30 µg/mL)	[35]
*O. caniculata*	Caxiuanã National Forest, Melgaço, PA	Leaf	β-selinene (20.30%), β-caryophyllene (18.90%), 7-*epi*-α-selinene (14.30%), and bicyclogermacrene (10.40%)	Antibacterial, microdilution method (*Escherichia coli,* MIC 19.50 µg/mL; *Staphylococcus epidermidis,* MIC 312.50 µg/mL; *Staphylococcus aureus*, MIC 625.0 µg/mL; *Bacillus cereus*, MIC 312.50 µg/mL), cytotoxic (MCF-7 mammary adenocarcinoma, IC_50_ 67.70 μg/mL)	[36]
*O. caudata*	Caxiuanã National Forest, Melgaço, PA	Leaf	Bicyclogermacrene (29.60%), germacrene D (19.90%), α-pinene (9.80%), and β-pinene (9.70%)	Antibacterial, microdilution method (*Escherichia coli,* MIC 19.50 µg/mL; *Staphylococcus epidermidis,* MIC 625.0 µg/mL; *Staphylococcus aureus*, MIC 625.0 µg/mL, *Bacillus cereus*, MIC 312.50 µg/mL), cytotoxic (MCF-7 mammary adenocarcinoma, IC_50_ 64.0 μg/mL)	[36]
*O. cujumary*	Caxiuanã National Forest, Melgaço, PA	Leaf	β-caryophyllene (22.20%), caryophyllene oxide (12.40%), 2-tridecanone (7.30%), and δ-cadinene (6.60%)	Antibacterial, microdilution method (*Escherichia coli,* MIC 19.50 µg/mL; *Staphylococcus epidermidis,* MIC 625.0 µg/mL; *Staphylococcus aureus*, MIC 625.0 µg/mL, *Bacillus cereus*, MIC 625.0 µg/mL), cytotoxic (MCF-7 mammary adenocarcinoma, IC_50_ 63.90 μg/mL)	[36]
*O. duckei*	Santa Rita, PB	Leaf, Steam bark, Fruits, and roots	Profile I: β-caryophyllene (60.54%), α-humulene (4.63%), δ-selinene (4.40%), and δ-cadinene (1.69%)	Cardiovascular (Wistar rats model) EO at 1.0, 5.0, 10.0 and 15.0 mg/kg. - Induced hypotension Leaves: (7.0, 15.0, 21.0 and 37.0%, respectively) Stem Bark: (8.0, 25.0, 38.0, 27.0%, respectively) Fruits: (6.0, 8.0, 18.0 and 26.0%, respectively) Roots: (4.0, 20.0, 33.0, 25.0%, respectively) - bradycardia leaves: (3.0, 9.0, 18.0 and 53.0%, respectively) Stem Bark: (5.0, 22.0, 53.0, 49.0%, respectively) Fruits: (3.0, 3.0, 12.0 and 35.0%, respectively) Roots: (3.0, 30.0, 57.0 and 35.0%, respectively)	[38]
			Stem Bark: β-eudesmol (27.51%), α-pinene (9.02%), limonene (6.65%), and borneol (6.18%)		
			Fruits: limonene (30.12%), β-pinene (12.25%), α-pinene (9.89%), and myrcene (7.86%);		
			Roots: elemol (24.31%), β-elemene (16.69%), β-eudesmol (13.44%), and borneol (3.69%)		
*O. elegans*	Restinga de Jurubatiba National Park, Carapebus, RJ	Leaf	Sesquirosefuran (92.2%)	Antiparasitic, *Rhipicephalus (Boophilus) microplus* (larval packet test [LPT], LC_50_ 59.68 mg/mL [24 h] and 25.59 mg/mL [48 h]; adult immersion test [AIT], LC_50_ 4.96 mg/mL and LC_90_ 17.37 mg/mL; larval repellency test [RT], LC_50_ 0.04 mg/mL and LC_90_ 1.24 mg/mL)	[51]
*O. gardneri*	Igarassu, PE	Leaf	Germacrene D (26.96%), bicyclogermacrene (20.73%), and viridiflorol (5.52%)	Acaricidal (*Tetranychus urticae*, 1.50 to 2.50 µL/cm^2^ of EO, percentages of repellency from 17.32% to 68%)	[40]
*O. gardneri*	not reported	Leaf	β-caryophyllene (29.28%), α-pinene (15.40%), kaurene (18.35%), and β-pinene (8.93%)	Molluscicidal (*Biomphalaria glabrata*, LC_90_ 16.50 mg/mL, LC_50_ 9.70 mg/mL, and LC_10_ 2.80 mg/mL)	[41]
*O. lancifolia*	Santa Maria, RS	Leaf	Seasonal study (fall): caryophyllene oxide (40.6%), *allo*-himachalol (8.0%), bulnesol (6.9%), bicyclogermacrene (6.1%)	Antifungal (*Fusarium moniliforme*, mycelial growth inhibition in 67.50% at 1.0 µL/mL)	[50]
*O. lancifolia*	Santa Maria, RS	Leaf	Seasonal study (fall): β-chenopodiol (20.9%), (*Z*)-nerolidyl acetate (9.3%), and caryophyllene oxide (7%)	Antifungal (*Fusarium moniliforme*, mycelial growth inhibition in around 50.0% at 1.0 µL/mL)	[50]
*O. lancifolia*	Santa Maria, RS	Inflorescences	Seasonal study: caryophyllene oxide (34.90%), bicyclogermacrene (8.10%), and atractylone (4.90%)	Antifungal (*Fusarium moniliforme*, mycelial growth inhibition in around 60.0% at 1.0 µL/mL)	[50]
*O. lancifolia*	Santa Maria, RS	Fruit	Seasonal study: caryophyllene oxide (42.10%), bicyclogermacrene (9.90%), and (*E*)-β-ocimene (3.10%)	Antifungal (*Fusarium moniliforme*, mycelial growth inhibition in around 62.0% at 1.0 µL/mL)	[50]
*O. nigrescens*	Manaus, AM	Leaf	β-caryophyllene (37.90%), β-pinene (6.90%), α-pinene (6.60%), linalool (5.50%), and α-copaene (6.20%)	Platelet aggregation activity (anti-aggregant factor with 10.80%)	[44]
*O. notata*	Carapebus, RJ	Leaf	β-caryophyllene (22.90%), germacrene A (22.70%), and α-pinene (8.70%)	Toxicological (*Artemia salina*, LC_50_ 2.37 μg/mL)	[42]
*O. odorifera*	Machado, MG	Leaf	Profile I: safrole (36.30%), γ-cadinene (6.60%), camphor (6.50%), and α-copaene (6.0%)	Antileishmanial (*Leishmania amazonensis*, amastigotes, IC_50_ 4,67 μg/mL), cytotoxic (mice BALB/c peritonal macrophages (CC_50_ 49.52 μg/mL)	[55]
*O. odorifera*	Marcelino Ramos, RS	Leaf	Profile II: camphor (43.0%), safrole (42.0%), camphene (6.0%), limonene (3.0%)	Insecticidal and repellent (maize weevil *Sitophilus zeamais*, LD_50_ 14.10 μL or 0.09 μL/cm^2^)	[53]
*O. odorifera*	Marcelino Ramos, RS	Leaf	Profile II: safrole (40.23%), camphor (34.35%), and limonene (7.42%)	Antibacterial, disc diffusion method: Gram-negative (*Acinetobacter* sp, *Aeromonas* sp, *Citrobacter freundii*, *Escherichia coli*, *Klebsiella pneumoniae, Proteus mirabilis, Proteus vulgaris, Salmonella choleraesuis, Serratia marcescens, Shigella flexneri, Yersinia enterocolitica*) and Gram-positive (*Enterococcus faecalis, Micrococcus luteus, Sarcina* sp*, Staphylococcus epidermidis, Streptococcus mutans, Staphylococcus aureus*), no MIC values reported; antioxidant, DPPH (IC_50_ 46.03 mg/mL)	[54]
*O. splendens*	Manaus, AM	Leaf	β-caryophyllene (51.0%), caryophyllene oxide (9.90%), α-humulene (6.20%)	Platelet aggregation activity (anti-aggregant factor with 11.74%)	[44]

**Legend:** TGI, anti-proliferative activity.
